# A Physiologically Based Pharmacokinetic and Pharmacodynamic (PBPK/PD) Model of Dapagliflozin in Type 2 Diabetes Mellitus: The Effect of Dosing, Hepatorenal Impairment, and Food

**DOI:** 10.3390/pharmaceutics18030287

**Published:** 2026-02-26

**Authors:** Nike Nemitz, Michelle Elias, Matthias König

**Affiliations:** 1 Faculty of Life Science, Institute for Biology, Systems Medicine of the Liver, Humboldt-Universität zu Berlin, Unter den Linden 6, 10099 Berlin, Germany; 2 Institute of Structural Mechanics and Dynamics in Aerospace Engineering, University of Stuttgart, Pfaffenwaldring 27, 70569 Stuttgart, Germany

**Keywords:** dapagliflozin, type 2 diabetes mellitus, physiologically based pharmacokinetic/pharmacodynamic model (PBPK/PD), pharmacokinetics, pharmacodynamics, personalized medicine

## Abstract

**Background/Objectives:** Dapagliflozin is an SGLT2 inhibitor prescribed for the management of type 2 diabetes mellitus. The drug lowers blood glucose levels by increasing urinary glucose excretion (UGE). Despite established efficacy, dapagliflozin demonstrates significant inter-individual variability in pharmacokinetics (PK) and pharmacodynamics (PD), with potential impact on treatment outcomes. **Methods:** To evaluate the sources of variability and to support patient stratification and model-informed individualized therapy, we developed a physiologically based pharmacokinetic/pharmacodynamic (PBPK/PD) model of dapagliflozin using curated data from 28 clinical studies. This framework integrates absorption, distribution, metabolism, excretion, and pharmacodynamics, and accounts for key determinants of variability including renal and hepatic function, and food effects. **Results:** The simulations reproduced dose-dependent pharmacokinetics with predicted C_max_ and AUC values typically within 10–15% of observed data. Renal impairment reduced UGE by 40–60% despite modest changes in plasma exposure, while hepatic impairment produced only small shifts in PK and PD. The model also reproduced the fed-state reduction of peak concentrations, consistent with the 30–50% decrease reported clinically. **Conclusions:** All model files, code, and curated datasets are openly available in line with FAIR standards and Open Science practices, enabling transparent and reproducible analyses and providing a mechanistic basis for individualized therapy in type 2 diabetes.

## 1. Introduction

Type 2 diabetes mellitus (T2DM) is a chronic metabolic disorder and a major global health challenge. Persistent hyperglycemia can lead to severe complications, and many patients do not achieve sustained glycemic control despite multiple available treatment options [1,2,3]. These challenges highlight the continued need for effective and well-characterized therapeutic strategies.

The kidney is central to glucose homeostasis by reabsorbing nearly all filtered glucose within the kidney’s proximal tubule. This process is mediated mainly by the sodium-glucose co-transporter 2 (SGLT2, 90%) and to a lesser extent by the sodium-glucose co-transporter 1 (SGLT1, 10%) [4,5]. Inhibition of SGLT2 lowers the renal threshold for glucose (RTG) and therefore increases urinary glucose excretion (UGE), ultimately reducing plasma glucose concentrations. This insulin-independent mechanism has been successfully used by various SGLT2 inhibitors (SGLT2i) [4].

Dapagliflozin, an SGLT2 inhibitor and the first drug of this class to receive approval, is widely used in the treatment of T2DM, chronic kidney disease, and heart failure. Clinically, dapagliflozin is typically prescribed at 5–10 mg once daily, with 10 mg being the standard maintenance dose. It is characterized by oral efficacy, high selectivity, and durable effects on glycated hemoglobin (HbA1c) [6,7]. The pharmacokinetics (PK) of dapagliflozin are well characterized. It is rapidly absorbed, reaching peak plasma concentrations within 2 h, has a half-life of approximately 13 h, and an oral bioavailability of about 78%. The drug is primarily metabolized by UDP glucuronosyltransferase family 1 member A9 (UGT1A9) to dapagliflozin-3-O-glucuronide (D3G), which accounts for the majority of urinary excretion, while unchanged dapagliflozin contributes less than 2% [7,8]. Pharmacodynamically, dapagliflozin produces dose-dependent increases in urinary glucose excretion that reflect its inhibitory effect on renal glucose reabsorption [9,10]. Patients with T2DM are at increased risk of developing comorbidities. Clinical studies have demonstrated that pathophysiological factors can significantly impact the pharmacokinetics and pharmacodynamics (PD) of dapagliflozin [9]. For example, renal impairment increases systemic exposure to dapagliflozin and its metabolite. At the same time, it reduces UGE and leads to diminished efficacy [11]. Hepatic impairment similarly increases exposure, raising potential safety concerns [9,12]. Furthermore, food–drug interaction studies consistently report delayed time to maximum plasma concentration and reduced C_max_ under fed conditions. Although these food–induced changes are often considered clinically negligible from a pharmacokinetic standpoint, as they do not affect overall exposure, they may still impact the pharmacodynamic effects of UGE, and therefore its efficiency as a drug. Together, these findings underscore the difficulty of predicting drug exposure and response in diverse patient populations [13,14,15,16].

Several computational modeling studies have examined different aspects of dapagliflozin pharmacology, including population pharmacokinetics and exposure-response analyses in special populations [7,17,18,19], meta-analytical PK/PD approaches [20], and physiologically based models focusing on pediatric dosing, SGLT inhibition, or drug-drug interactions [21,22,23]. Additional mechanistic, quantitative systems pharmacology, and translational modeling approaches have also been reported [24,25,26,27,28]. While each provided valuable insights, they typically focus on specific sub-questions in isolation rather than offering an integrated view of dapagliflozin pharmacokinetics and pharmacodynamics.

A major limitation of existing dapagliflozin models is their limited reusability and reproducibility. Most models are not openly available: the underlying software is closed, executable code is not provided, curated clinical datasets are inaccessible, and models and workflows do not follow FAIR principles. As a consequence, independent reproduction, long–term preservation, and systematic extension of these models are not ensured. In addition, many existing models rely on restricted or narrowly defined datasets, limiting their applicability across patient populations and clinical scenarios. Together, these limitations severely hamper reuse, cross–validation, and cumulative model development. A systematic overview of existing dapagliflozin models, including their scope, availability, and reproducibility, is provided in Supplementary Table S1.

Reproducibility is a broader challenge in systems biology [29] and PBPK modeling more generally [30], with many published models lacking accessible equations, data, or executable workflows. To address these challenges, we developed the present model in accordance with FAIR principles [31] and Open Science practices, ensuring that all model files, simulation code, and curated datasets are openly available and reusable.

Physiologically based pharmacokinetic/pharmacodynamic (PBPK/PD) modeling provides a quantitative framework to integrate physiological, biochemical, and drug-specific data. By representing the processes of absorption, distribution, metabolism, and excretion (ADME) in mechanistic detail, such models can simulate the impact of organ dysfunction, prandial state, and other patient-specific factors on exposure and response, offering insights beyond what can be obtained from clinical trials alone [32,33,34].

Here, we present a fully reproducible and openly available PBPK/PD model of dapagliflozin, encoded in SBML and developed using published clinical data from healthy individuals, patients with type 2 diabetes mellitus, and individuals with renal or hepatic impairment. In contrast to earlier dapagliflozin models that addressed isolated aspects, our unified whole–body framework simultaneously integrates dose dependency, renal and hepatic dysfunction, glucose dependency, and food effects within a single mechanistic system. The model was calibrated and evaluated across multiple datasets and subsequently applied to quantify variability in dapagliflozin pharmacokinetics and pharmacodynamics under clinically relevant scenarios. By combining whole–body integration with full transparency and open access to model equations, code, and curated datasets, this work provides a reproducible mechanistic foundation for studying variability in dapagliflozin exposure and response and supports model–informed dose recommendations.

## 2. Materials and Methods

The computational model of dapagliflozin was developed as a PBPK/PD model through a systematic workflow combining clinical data curation, mechanistic modeling, and in silico simulations. This involved a structured literature search, development of an SBML-based model, parameter optimization with selected data subset, and simulation experiments reflecting clinical trial conditions. Pharmacokinetic and pharmacodynamic outcomes were then analyzed to characterize drug disposition and variability across physiological, pathophysiological and prandial states.

### 2.1. Systematic Literature Research and Data Curation

A systematic literature search was conducted to compile studies that reported pharmacokinetic and pharmacodynamic data of dapagliflozin. PubMed was queried on 23 April 2024 using the terms dapagliflozinAND pharmacokinetics, and the PKPDAI (https://www.pkpdai.com/, accessed on 23 April 2024) database was screened in parallel [35]. The clinical studies encompassed a diverse cohort of participants, including healthy volunteers, patients diagnosed with type 1 or type 2 diabetes, and studies investigating renal or hepatic impairment. Pediatric studies, animal studies, and reports lacking sufficient data were excluded from the analysis. The Supplementary Materials Figure S1 provides an overview of the literature review process. Data from eligible studies were curated in the open pharmacokinetics database PK-DB (https://pk-db.com, accessed on 17 February 2026) [36]. Information on demographics, disease status, dosing protocols, plasma and urine concentration-time profiles of dapagliflozin and D3G, and pharmacodynamic outcomes such as urinary glucose excretion were extracted according to established protocols [36]. Figure-based data were digitized using WebPlotDigitizer (https://automeris.io/, accessed on 17 February 2026) [37], while tabular and textual data were reformatted into standardized PK-DB formats [36]. The curated dataset, including patient and study characteristics, dosing regimens, and PK/PD outcomes, provided the basis for PBPK/PD model development and is publicly available via PK-DB (https://pk-db.com, accessed on 17 February 2026). Table 1 provides an overview of the curated studies, along with the PK-DB IDs for each study.

### 2.2. Computational Model

The PBPK/PD model was developed in the Systems Biology Markup Language (SBML) [58,59]. Programmatic model construction and visualization were performed using the sbmlutils [60] and cy3sbml [61,62] libraries. Numerical solutions of the underlying ordinary differential equations (ODEs) were obtained with sbmlsim [63], powered by the high-performance SBML simulation engine libroadrunner [64,65]. The complete model, including simulation scripts and documentation, is available in SBML format under a CC-BY 4.0 license via GitHub with version 0.9.8 used for the analysis (https://github.com/matthiaskoenig/dapagliflozin-model, accessed on 17 February 2026) and archived on Zenodo 0.9.8 (https://doi.org/10.5281/zenodo.18011516) [66].

**Figure 1:**
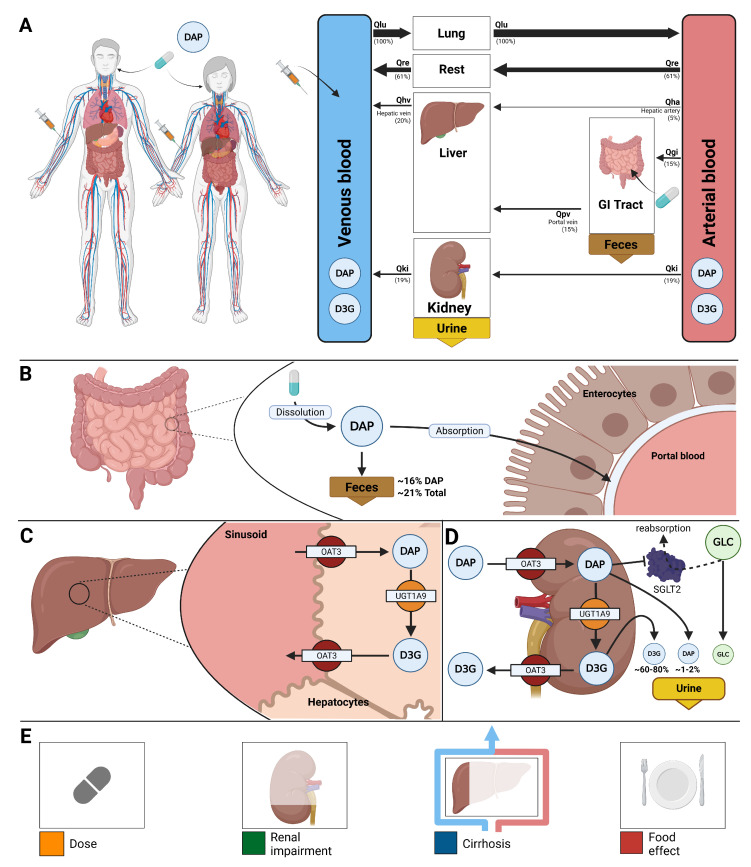
Whole-body PBPK/PD model of dapagliflozin and key factors influencing its disposition.(**A**) Whole-body model showing dapagliflozin (DAP) administration (oral and intravenous), its systemic circulation, and the key organs (liver, kidney, GI tract) involved in metabolism, distribution, and excretion. (**B**) Intestine model illustrating DAP absorption by enterocytes; 16% is excreted in feces. (**C**) Hepatic model showing DAP uptake by hepatocytes and conversion to its primary metabolite D3G via UGT1A9. (**D**) Renal model showing uptake and excretion of DAP and D3G in urine and metabolic conversion of dapagliflozin to D3G. DAP inhibits SGLT2, reducing glucose reabsorption and increasing urinary glucose excretion. (**E**) Key factors influencing DAP disposition included in the model: administered dose, renal impairment, liver function (cirrhosis), and food effects (absorption activity).

The model comprises a whole-body framework with submodels for the intestine, liver, and kidney, linked through the systemic circulation (Figure 1) to characterize dapagliflozin’s absorption, distribution, metabolism, and excretion. The model has a hierarchical structure, with the whole-body model linking the submodels. Key ADME processes modeled include intestinal absorption (first-order uptake from the gastrointestinal tract), hepatic and renal glucuronidation by UGT1A9 to form the major metabolite D3G, tissue distribution, renal filtration, and urinary and fecal excretion of dapagliflozin and D3G. The pharmacodynamic component linked dapagliflozin plasma concentrations to UGE through inhibition of renal glucose reabsorption, parameterized by fasting plasma glucose and RTG. Mathematical descriptions of the submodels and ODEs are provided in the Supplementary Materials Sections S3 and S4.

Fractional organ volumes and blood flows were taken from literature sources [32]. The fractional compartment volumes were set to $FV_{\mathrm{gu}}=1.71\%$ for the gut, $FV_{\mathrm{ki}}=0.44\%$ for the kidneys, $FV_{\mathrm{li}}=2.10\%$ for the liver, and $FV_{\mathrm{lu}}=0.76\%$ for the lungs. Fractional blood flows were defined as $FQ_{\mathrm{gu}}=18.00\%$ for the gut, $FQ_{\mathrm{ki}}=19.00\%$ for the kidneys, $FQ_{h}=21.50\%$ for the hepatic venous outflow, and $FQ_{\mathrm{lu}}=100\%$ for the lungs. Absolute organ volumes and blood flows were calculated by scaling the corresponding fractional values with body weight.

Tissue-to-plasma partition coefficients were assumed to be identical across all tissues for dapagliflozin, with a fixed value of $Kp^{\mathrm{DAP}}=25.52$. No tissue partitioning was assumed for the metabolite D3G. Transport processes of dapagliflozin and D3G in the liver and kidneys were modeled explicitly.

Several key factors influencing pharmacokinetic and pharmacodynamic variability were implemented as scaling parameters. Renal impairment was modeled as a progressive decline in renal function using the factor f_renal_, which was applied to glomerular filtration rate (GFR) and renal clearance of dapagliflozin and D3G. Scaling values were mapped from KDIGO categories: normal (eGFR ≥ 90 mL/min, $f_{\mathrm{renal}}=1.00$), mild (GFR 50–89 mL/min, $f_{\mathrm{renal}}=0.69$), moderate (GFR 30–49 mL/min, $f_{\mathrm{renal}}=0.32$), severe (GFR ≤30 mL/min, $f_{\mathrm{renal}}=0.19$), derived from KDIGO guidelines and related modeling studies [67,68]. Hepatic impairment was modeled using the scaling factor f_cirrhosis_, representing reduced functional liver parenchyma and blood shunting. Scaling values were mapped to Child–Turcotte–Pugh classes: A (mild: 5–6 points, f_cirrhosis_ = 0.40), B (moderate: 7–9 points, f_cirrhosis_ = 0.70), and C (severe: 10–15 points, f_cirrhosis_ = 0.80) [69,70,71,72]. Prandial effects were incorporated via the intestinal absorption scaling factor f_absorption_ (fasted: 1.00, fed: 0.30). The given dose can be modified by changing the parameters corresponding to the intravenous and oral doses of dapagliflozin, IVDOSE_dap_ and PODOSE_dap_, respectively. Subject- and study-specific physiological and clinical parameters were incorporated when available, including bodyweight, glomerular filtration rate to adjust renal function, and fasting plasma glucose for the pharmacodynamic component.

Multiple dosing regimes were implemented by stepwise numerical integration between dosing intervals, with dosing parameters IVDOSE_dap_ and PODOSE_dap_ set according to the respective dosing protocol of the study. All simulations were demeterministic with the mean model corresponding to the optimized parameters. No population variability was included.

Multiple-dose regimens were implemented by stepwise numerical integration between dosing intervals, with dosing events applied according to the study-specific protocols. Oral and intravenous doses were specified using the parameters PODOSE_dap_ and IVDOSE_dap_, respectively. Simulation time horizons and post-dose sampling windows were selected to match the corresponding clinical study designs.

All simulations were performed deterministically using the optimized parameter set representing the typical (mean) individual. Inter-individual or between-subject variability was not included, as the objective was to evaluate typical pharmacokinetic and pharmacodynamic behavior across studies rather than to perform population-based variability analyses.

The Physiome Journal [73] has demonstrated the reproducibility, reusability, and discoverability of the mathematical model and computational simulations.

### 2.3. Model Assumptions

Additional details on model equations and assumptions are provided in Supplementary Material S4. The key model assumptions and simplifications are summarized below.

- Dapagliflozin absorption was modeled as a first-order process.
- Diurnal variation in plasma glucose concentrations was not modeled explicitly. Instead, a constant fasting plasma glucose (FPG) concentration was assumed and used for the calculation of urinary glucose excretion (UGE). When reported, study-specific FPG values were used. Otherwise, FPG values of 5 mM for healthy subjects, 7.5 mM for subjects with type 1 diabetes mellitus (T1DM), and 7.5 mM for subjects with type 2 diabetes mellitus (T2DM) were assumed.
- The renal glucose threshold (RTG) was parameterized using parameter optimization, with optimized values reported in Supplementary Table S3.
- Renal filtration and tubular glucose reabsorption were not modelled explicitly. Renal elimination of dapagliflozin (DAP) and its metabolite D3G was instead described using first-order processes, depending on kidney volume, renal function (glomerular filtration rate), and compound-specific excretion rate constants. The parameters KI__DAPEX_k and KI__D3GEX_k were estimated via parameter optimization.
- The conversion of DAP to D3G by UGT1A9 in the liver and kidneys was modelled using irreversible Michaelis–Menten kinetics. The Michaelis constant was fixed to the value reported from kidney microsome experiments for dapagliflozin ($K_{m}=479\;\mu M$ [11]). The corresponding maximum reaction rates were estimated by parameter optimization (DAP2D3G_Vmax and KI__f_DAP2D3G).
- All model parameters not estimated via parameter optimization were taken from literature sources, with the exception of transport-related parameters. Transport processes for DAP and D3G in the liver and kidneys were assumed to be fast and reversible relative to metabolic conversion and were therefore not rate-limiting.
- Food effects were incorporated by assuming a reduction in the absorption rate constant in the fed state. Specifically, the absorption rate was reduced by 70% relative to the fasted state (from 1.0 to 0.3). This assumption was motivated by delayed gastric emptying following a standard high-fat meal, which can increase gastric emptying time from less than 30 min in the fasted state to approximately 100 min or more. The reduction in absorption rate was assumed to be proportional to the change in gastric emptying time, resulting in a fed-state absorption factor of
  $$f_{\mathrm{absorption},\;\mathrm{fed}}=\frac{30}{100}=0.3.$$

### 2.4. Model Parameterization

A subset of curated data from healthy subjects, patients with type 2 diabetes mellitus, and individuals with renal impairment following single–dose administration was used for parameter optimization. Model parameters were estimated using a local optimization approach, and the resulting optimal parameter set was applied consistently across all subsequent simulations without further study–specific refitting.

The cost function, defined as a function of the parameter vector $\vec{p}$, minimized the sum of squared, weighted residuals $r_{i,k}$ across all time courses *k* and data points *i*. Time courses were weighted by the number of participants in each study $n_{k}$, and individual time points were weighted by the inverse of the associated measurement uncertainty, represented by the standard deviation $\sigma_{i,k}$. This resulted in weights $w_{i,k}=n_{k}/\sigma_{i,k}$.

$$F\left(\vec{p}\right)=\frac{1}{2}\sum_{i,k}{\left(w_{i,k}\cdot r_{i,k}\left(\vec{p}\right)\right)}^{2}$$

To mitigate sensitivity to initial conditions, multiple optimization runs (n=100) were performed using different initial parameter values. Optimization was conducted sequentially: pharmacokinetic parameters were estimated first (Table S2), followed by pharmacodynamic parameters (Table S3). All optimized parameter values and the corresponding parameter bounds used during optimization are reported in Supplementary Materials, Section S5.

### 2.5. Simulations

For each curated clinical study (Table 1), a corresponding in silico experiment was implemented to reproduce the reported dosing regimen and study conditions. Where available, mean demographics were applied; otherwise, standard references were used. Parameters for oral and intravenous dosing, prandial state, bodyweight, fasting plasma glucose (FPG), and renal or hepatic function were adjusted according to study-specific information. Multiple dosing protocols were incorporated where applicable. To further explore sources of variability, simulation experiments and parameter scans were performed across physiologically relevant ranges for renal function, hepatic function, intestinal absorption activity, and dose to enable systematic evaluation of the influence of key physiological and pathophysiological parameters on PK/PD outcomes.

### 2.6. Sensitivity Analysis

The impact of model parameters on pharmacokinetic and pharmacodynamic outcomes was evaluated using sensitivity analysis. A reference simulation of a single oral dose of 10 mg dapagliflozin was used. Pharmacokinetic readouts included area under the concentration–time curve (AUC), maximum concentration ($C_{\max}$), and half-life for dapagliflozin and its metabolite D3G, while urinary glucose excretion (UGE) at 24 h served as the pharmacodynamic readout.

Parameters representing physical constants, conversion factors, and dosing were excluded from the analysis. Normalized sensitivities with absolute values below 0.1, as well as parameters without measurable effects on any readout, were omitted from the visualization. For clarity, sensitivity matrices were hierarchically clustered by model parameters using the single linkage method.

Local sensitivities were computed by perturbing each parameter $p_{i}$ individually by ±1% relative to its reference value $p_{i,0}$. Sensitivities were calculated using a symmetric midpoint approximation,

$$S\left(q_{k},p_{i}\right)=\frac{q_{k}\left(p_{i}^{+}\right)-q_{k}\left(p_{i}^{-}\right)}{p_{i}^{+}-p_{i}^{-}},$$

where $p_{i}^{\pm }=p_{i,0}\cdot \left(1\pm 0.01\right)$. Sensitivities were then normalized to obtain dimensionless measures,

$$S_{\mathrm{norm}}\left(q_{k},p_{i}\right)=\frac{q_{k}\left(p_{i}^{+}\right)-q_{k}\left(p_{i}^{-}\right)}{p_{i}^{+}-p_{i}^{-}}\cdot \frac{p_{i,0}}{q_{k}\left(p_{i,0}\right)},$$

representing the relative change in the output per relative change in the parameter.

### 2.7. Pharmacokinetic and Pharmacodynamic Parameters

Pharmacokinetic parameters of dapagliflozin and D3G were calculated from plasma concentration-time curves and urinary excretion using standard non-compartmental methods. Pharmacodynamic outcomes were evaluated in terms of UGE, calculated from the simulated plasma concentration-time courses in combination with fasting plasma glucose and the renal threshold for glucose. Simulated profiles and derived pharmacokinetic and pharmacodynamic parameters were compared against the curated clinical data for dapagliflozin and its primary metabolite D3G.

## 3. Results

### 3.1. Dapagliflozin Database

An extensive database of dapagliflozin’s pharmacokinetics and pharmacodynamics was created to develop and validate the model. A systematic literature search initially yielded 190 records. After screening according to predefined inclusion and exclusion criteria, 28 studies were selected for detailed curation and formed the core dataset used to evaluate the PBPK/PD model. See Supplementary Materials Figure S1 for the study selection process. The complete Zotero literature library is publicly available at https://www.zotero.org/groups/6355063/dapagliflozin-model/library (accessed on 17 February 2026). All curated pharmacokinetic and pharmacodynamic data of the 28 studies are publicly available in the model files and in the PK-DB (https://pk-db.com, accessed on 17 February 2026) database with unique study identifiers as referenced in the manuscript (Table 1).

### 3.2. Computational Model

Using the curated dataset, a physiologically based pharmacokinetic/pharmacodynamic model was developed to describe the absorption, distribution, metabolism, and elimination (ADME) of dapagliflozin and its primary metabolite, D3G, as well as its pharmacodynamic effect on UGE (Figure 1). The complete model, including simulation scripts and documentation, is available in SBML format under a CC-BY 4.0 license via GitHub (https://github.com/matthiaskoenig/dapagliflozin-model, accessed on 17 February 2026) and archived on Zenodo 0.9.8 (https://doi.org/10.5281/zenodo.18011516) [66].

Simulations followed the respective clinical study designs, accounting for single- and multiple-dose regimens, prandial state, and renal or hepatic impairment. Additional simulations are provided in Supplementary Materials Section S5 (Figures S6–S21).

The final parameter set is summarized in the Supplementary Materials Tables S2 and S3. Parameter convergence (R^2^, RMSE) and goodness-of-fit metrics are shown in the Supplementary Materials Figure S5. All submodel visualizations are provided in Supplementary Materials Section S2 (Figures S2–S4), and all model equations and ODEs are given in Supplementary Materials Section S4. The sensitivity analysis results are reported in Supplementary Section S7.

### 3.3. Dose Dependency

**Figure 2:**
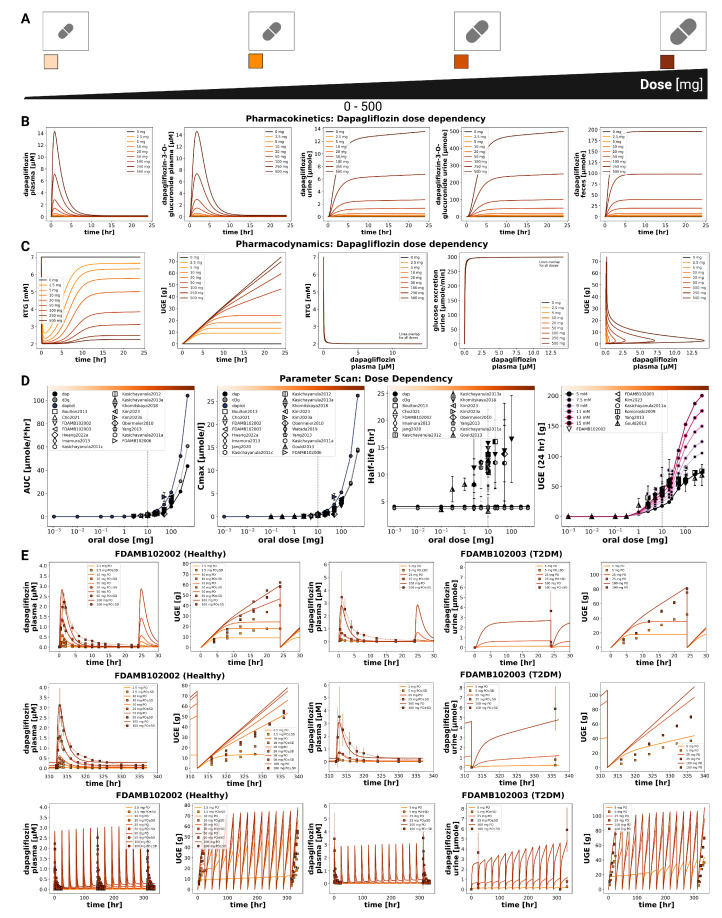
Dose-dependent pharmacokinetics and pharmacodynamics of dapagliflozin.(**A**) Oral dose range (0–500 mg). (**B**) Pharmacokinetic time courses of dapagliflozin and D3G in plasma, urine, and feces. (**C**) Pharmacodynamic time courses showing RTG and UGE, and exposure-response relationships. (**D**) Pharmacokinetic parameters ($AUC_{\inf}$, $C_{\max}$, and half-life) for dapagliflozin and D3G, and 24 h UGE; observed parameters overlaid where available. (**E**) Comparison of simulations with study data FDAMB102002 [40] and FDAMB102003 [41]. Simulations are shown as solid lines and study data as symbols with SDs where available.

**Figure 3:**
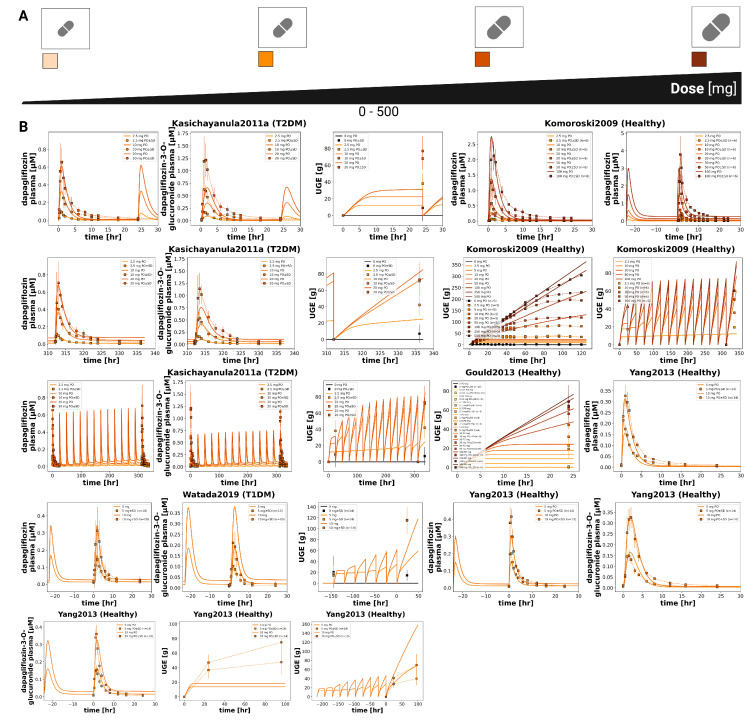
Additional dose-dependent clinical studies of dapagliflozin. (**A**) Oral dose range (0–500 mg). (**B**) Comparison of simulations with study data Gould2013 [44], Kasichayanula2011a [48], Komoroski2009 [14], Watada2019 [56], and Yang2013 [57]. Simulations are shown as solid lines, and study data are shown as dashed lines with squares and SDs where available.

The pharmacokinetic and pharmacodynamic effects of dapagliflozin over an oral dose range of 0–500 mg are shown in Figure 2 and Figure 3. With increasing doses, plasma concentrations of dapagliflozin and its primary metabolite D3G increased, as did the amounts excreted in urine and feces. Dapagliflozin reached peak plasma levels after approximately 3 h and returned to baseline within approximately 10 h, whereas D3G showed a slightly delayed peak and elimination. The parameter scan showed a clear dose-dependent rise in exposure metrics (AUC and $C_{\max}$), while half-lives remained largely unchanged. Higher doses of dapagliflozin were associated with lower RTG and a nonlinear increase in urinary glucose excretion.

Time-course simulations were performed for all curated clinical dose-dependency studies (FDAMB102002 [40], FDAMB102003 [41], Kasichayanula2011a [48], Komoroski2009 [14], Gould2013 [44], Watada2019 [56], Yang2013 [57]). Simulated dapagliflozin and D3G plasma concentrations, urinary excretion, and UGE are shown for both single dose and multiple dose regimens.

In addition to dose-dependency, glucose-dependent simulations were performed to explore how varying plasma glucose levels influence pharmacokinetic and pharmacodynamic outcomes (Supplementary Figure S21). Pharmacokinetics remained unchanged across glucose concentrations from 3 to 15 mM, while higher glucose levels were associated with higher UGE.

### 3.4. Renal Impairment

**Figure 4:**
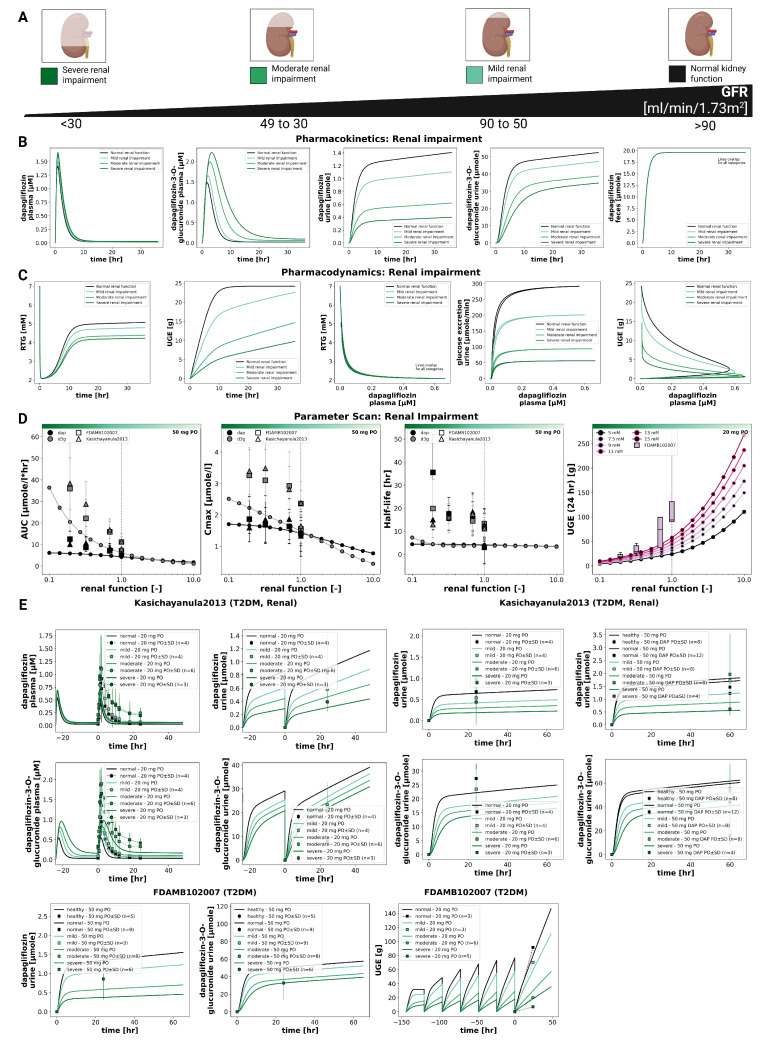
Effect of renal impairment on pharmacokinetics and pharmacodynamics of dapagliflozin. (**A**) Renal function categories from normal to severe impairment used in simulations. (**B**) Pharmacokinetic time courses of dapagliflozin and D3G in plasma, urine, and feces following a 50 mg dose. (**C**) Pharmacodynamic time courses showing RTG, UGE, and exposure-response relationships, following a 20 mg dose. (**D**) Pharmacokinetic parameters ($AUC_{\inf}$, $C_{\max}$, and half-life) for dapagliflozin and D3G following a 50 mg dose, and UGE (24 h) following a 20 mg dose. (**E**) Comparison of simulations with study data Kasichayanula2013 [11] and FDAMB102007 [43]. Simulations are shown as solid lines, and study data as symbols with SDs where available.

The impact of renal impairment on dapagliflozin disposition and pharmacodynamics is shown in Figure 4. Simulations were performed for four renal function groups (normal, mild, moderate, severe). Plasma concentrations of dapagliflozin were minimally affected by renal dysfunction, whereas exposure to its main metabolite (D3G) increased with declining renal function. Urinary excretion of both dapagliflozin and D3G decreased with impairment, while fecal excretion remained unchanged. Across renal function states, parent AUC and $C_{\max}$ changed modestly, while D3G exposure increased with impairment.

The pharmacodynamic response showed a reduction in UGE with declining renal function, while RTG changed slightly. In severe impairment, simulated UGE was clearly lower than under normal function, whereas RTG was only minimally affected.

Simulated parent and metabolite plasma time courses and urinary excretion profiles across renal function groups are shown together with clinical data from Kasichayanula2013 [11] and FDAMB102007 [43]. Fecal excretion data were not available for direct comparison.

### 3.5. Hepatic Impairment

**Figure 5:**
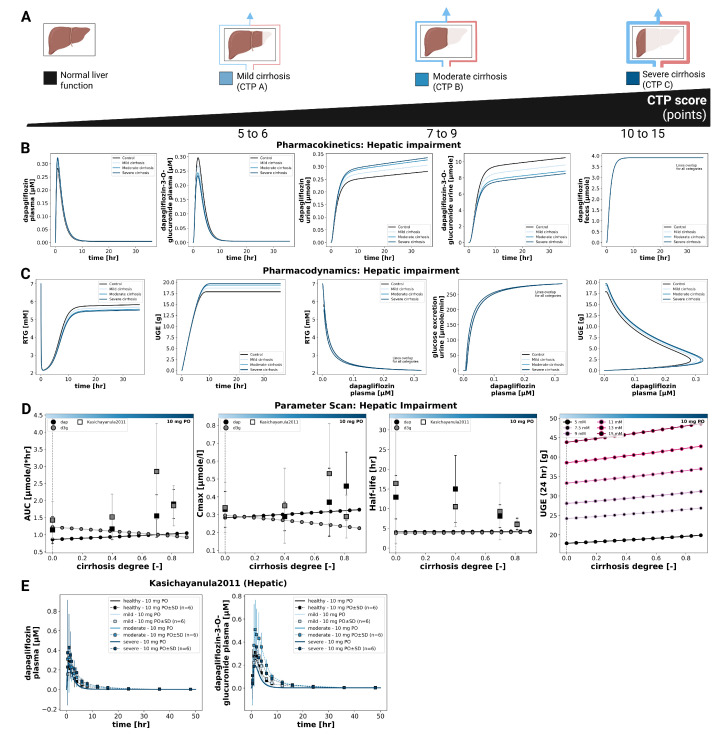
Effect of hepatic impairment on pharmacokinetics and pharmacodynamics of dapagliflozin. (**A**) Liver function categories used in simulations. (**B**) Pharmacokinetic time courses of dapagliflozin and D3G in plasma, urine, and feces across different CTP stages following a 10 mg dose. (**C**) Pharmacodynamic time courses showing RTG and UGE, and exposure-response relationships following a 10 mg dose. (**D**) Pharmacokinetic parameters ($AUC_{\inf}$, $C_{\max}$, and half-life) for dapagliflozin and D3G, and 24 h UGE, across cirrhosis degrees following a 10 mg dose. (**E**) Comparison of simulations with study data from Kasichayanula2011 [9]. Simulations are shown as solid lines, and study data as symbols with SDs where available.

The effect of hepatic impairment on dapagliflozin pharmacokinetics and pharmacodynamics is summarized in Figure 5. Simulations were performed for normal liver function and for mild, moderate, and severe cirrhosis. Plasma time courses of dapagliflozin and D3G were similar across groups. With increasing severity, dapagliflozin plasma exposure showed small increases in $C_{\max}$ and AUC, whereas exposure of the main metabolite D3G showed small decreases. Urinary excretion showed the same inverse pattern for dapagliflozin and D3G, while fecal excretion was unchanged. Parameter scans showed that half-life changed only slightly for both compounds.

Pharmacodynamic changes were limited. UGE increased slightly with cirrhosis severity, and RTG decreased slightly. UGE remained glucose dependent at all hepatic function states, and pharmacokinetics were unaffected by glycemia.

Simulated parent and metabolite plasma concentrations and urinary excretion profiles under different hepatic function states are shown together with clinical data from Kasichayanula2011 [9].

### 3.6. Food Effects

**Figure 6:**
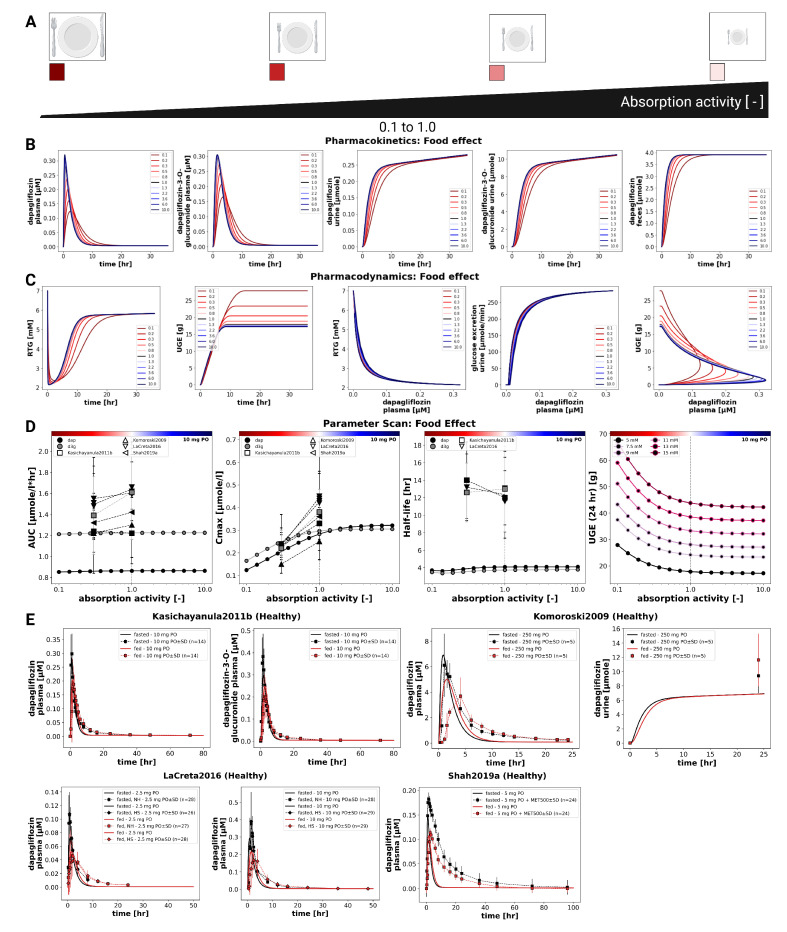
Effect of food on pharmacokinetics and pharmacodynamics of dapagliflozin. (**A**) Prandial states represented by changes in intestinal absorption activity. (**B**) Pharmacokinetic time courses of dapagliflozin and D3G in plasma, urine, and feces following a 10 mg dose. (**C**) Pharmacodynamic time courses showing RTG and UGE, and exposure-response relationships following a 10 mg dose. (**D**) Pharmacokinetic parameters ($AUC_{\inf}$, $C_{\max}$, and half-life) for dapagliflozin and D3G, and 24 h UGE, across absorption activities following a 10 mg dose. (**E**) Comparison of simulations with study data from Kasichayanula2011b [13], Komoroski2009 [14], LaCreta2016 [15], and Shah2019a [16] under fed and fasted conditions. Simulations are shown as solid lines, and study data as symbols with SDs where available.

The influence of food intake on dapagliflozin pharmacokinetics and pharmacodynamics was evaluated by systematically varying the fractional absorption parameter over a range from 0.1 to 10.0 (Figure 6). Simulations were conducted for a single 10 mg oral dose. Lower absorption activity resulted in slower and smaller increases in plasma concentrations of dapagliflozin and D3G, as well as slightly slower urinary excretion of both compounds. Pharmacokinetic analysis showed that changes in absorption predominantly affected $C_{\max}$, while AUC and half-life remained almost unchanged.

Pharmacodynamic simulations showed that higher absorption activity produced a larger magnitude of UGE, whereas lower absorption led to delayed and reduced glucosuria.

In clinical studies under fasted and fed conditions (Kasichayanula2011b [13], Komoroski2009 [14], LaCreta2016 [15], Shah2019a [16]), food intake reduced $C_{\max}$ by around 30–50%. Direct UGE comparisons between fed and fasted conditions were not available in these studies.

## 4. Discussion

In this study, we established a comprehensive clinical dataset of dapagliflozin pharmacokinetics and pharmacodynamics and used it to develop a mechanistic PBPK/PD model. In total, 28 clinical trials were curated, covering a broad spectrum of dosing regimens, prandial states, and populations with renal and hepatic impairment. While data availability was generally sufficient for model development, notable gaps remain. Quantitative information on fecal excretion originates from a single study, RTG measurements are available for only one study, and UGE under fed conditions is sparsely reported. These gaps highlight the need for more systematic reporting of PD endpoints and excretion routes in clinical pharmacology studies.

The PBPK/PD framework integrates intestinal absorption, hepatic metabolism, and renal clearance into a coherent representation of dapagliflozin disposition and effect. Robust parameter fitting and overall good agreement with observed data across studies support the reliability of the model. A distinct advantage of this mechanistic approach is the ability to investigate scenarios not easily addressed in clinical trials, such as direct comparisons of hepatic versus renal impairment or systematic exploration of absorption variability. Furthermore, pharmacodynamics are driven by total plasma concentrations via SGLT2 inhibition that modulates the RTG, providing a physiologically interpretable link from exposure to UGE.

The simulations reproduced the expected dose–response behavior, as increasing doses led to higher plasma concentrations and greater UGE, while half–life remained largely unchanged. Glucose–dependency analysis confirmed that pharmacokinetics were independent of plasma glucose in the tested range, whereas pharmacodynamics showed strong glucose dependence, consistent with greater UGE when plasma glucose exceeds RTG. These results align with clinical observations of increased glucosuria in hyperglycemic states.

Evaluation of organ dysfunction highlighted important differences between hepatic and renal impairment. Hepatic impairment produced only minor changes in parent exposure and small pharmacodynamic effects, despite measurable shifts in the metabolite. In contrast, renal impairment markedly increased D3G exposure and substantially reduced UGE, reflecting the central role of the kidneys in both metabolite elimination and the pharmacodynamic endpoint. Together, these findings emphasize that renal function is the dominant determinant of variability in both D3G disposition and UGE under the conditions studied.

The analysis of food effects highlights the importance of distinguishing between the extent and the rate of absorption. Although systemic exposure (AUC) remained essentially unchanged under fed conditions, both simulations and clinical data demonstrated a consistent reduction in C_max_ (approximately 30–50%). The model further indicated that these changes can diminish UGE, revealing a pharmacodynamic consequence that may be overlooked when focusing solely on AUC ratios. Changes in the absorption rate also influenced the timing and extent of glucosuria, with higher absorption activity producing earlier and larger UGE responses, whereas slower absorption resulted in delayed and reduced UGE. These simulation findings were consistent with clinical observations under fed and fasted conditions. Given the limited PD datasets under fed/fasted conditions, the food effect on dapagliflozin may warrant closer consideration when therapeutic goals depend on peak SGLT2 inhibition and UGE magnitude.

Some aspects remain insufficiently characterized. Data are incomplete for RTG measurements, fecal excretion, and UGE under fed conditions and in hepatic impairment. In addition, unexpected patterns in D3G concentrations during moderate hepatic impairment were observed and could reflect a combination of reduced UGT1A9 capacity, altered hepatic blood flow/shunting, or changes in renal handling. The current model represents typical physiology for each study population and scenario, but it only includes limited ways to account for individual differences. Variability in physiological parameters between subjects is not explicitly modeled, so the framework describes typical exposure and response rather than full population-level dispersion. Intestinal metabolism was not modeled as a separate mechanistic pathway. First-pass processes were captured effectively, which is consistent with the literature identifying hepatic and renal metabolism as the dominant clearance routes for dapagliflozin. However, if intestinal glucuronidation contributes more than is currently assumed, it could affect the predicted extent and timing of first-pass metabolism. Validation of fed-state UGE remains limited because clinical studies report pharmacokinetic outcomes but not corresponding pharmacodynamic measurements. Finally, several datasets required digitization from published figures, and although quality checks were applied, small errors cannot be completely excluded.

Future work should prioritize prospective clinical datasets with standardized UGE and RTG measurements under fed and fasted conditions, larger cohorts with renal and hepatic impairment, and quantitative characterization of hepatic glucuronidation capacity. Incorporating drug-drug interaction data and extending the framework to account for inter-individual variability could further improve individualized dosing strategies. More comprehensive data on fecal elimination would also strengthen the model.

Beyond its pharmacological insights, this work addresses a broader and well-recognized challenge in computational pharmacology and systems biology. Many published PBPK models remain difficult or impossible to reproduce because model files, equations, simulation code, and the datasets used for calibration and validation are often not publicly accessible [29,30]. This lack of transparency limits independent verification and substantially hinders reuse, extension, and cumulative model development by the wider community.

In contrast, the present study places reproducibility and accessibility at its core. The entire modeling framework—including the SBML model, simulation code, and curated clinical datasets—is openly available and explicitly designed to comply with FAIR principles [31,74]. By enabling independent reproduction and systematic reuse, this work facilitates transparent model evaluation and supports the long-term development of interoperable PBPK/PD models. In contrast to the only other accessible dapagliflozin PBPK model [24], which is distributed under a GPL-2.0 license, all models and resources presented here are released under permissive MIT and CC-BY licenses. This choice substantially lowers barriers to reuse, allowing straightforward integration into existing academic and industrial code bases, as well as enabling commercial application and downstream translation of the model.

In conclusion, the present PBPK/PD model integrates diverse clinical data into a mechanistic framework that captures key pharmacokinetic and pharmacodynamic features across patient populations and clinical scenarios. The model provides insight into dose dependency, glucose dependency, food–drug interactions, and organ impairment, and can inform dose recommendations by quantifying the sources and magnitude of variability in drug response. By providing full open access to the model, simulation code, and curated datasets, this work establishes a transparent and reproducible reference framework that facilitates independent validation, reuse, and future PBPK/PD model development.

## Figures and Tables

**Table 1:** Summary of studies for modeling. Overview of study identifiers, PK-DB IDs, administered substance, route, dosing, and subject characteristics, including health status (H), renal impairment (RI), hepatic impairment (HI), fasting status and urinary glucose excretion (UGE) and renal threshold for glucose (RTG). DAP P = dapagliflozin plasma, DAP U = dapagliflozin urine, DAP F = dapagliflozin feces, D3G P = dapagliflozin-3 glucuronide plasma, D3G U = dapagliflozin-3 glucuronide urine. ✓ = respective data in study.

Study	PK-DB	Substance	Route	Dosing	Dose [mg]	H	RI	HI	T1	T2	DAP P	DAP U	DAP F	D3G P	D3G U	Fed	Fast	UGE	RTG
Boulton2013 [38]	PKDB00838	dap, [14C]dap	oral, IV	single	10, 0.080	✓					✓								
Cho2021 [39]	PKDB00839	dap	oral	single	10	✓					✓								
FDAMB102002 [40]	PKDB00959	dap	oral	multi	2.5, 10, 20, 50, 100	✓					✓							✓	
FDAMB102003 [41]	PKDB00960	dap	oral	single, multi	5, 25, 100					✓	✓							✓	
FDAMB102006 [42]	PKDB00970	[14C]dap	oral	single	50	✓					✓	✓	✓		✓				
FDAMB102007 [43]	PKDB00971	dap	oral	single, multi	20, 50	✓	✓			✓	✓	✓							
Gould2013 [44]	PKDB00840	dap	oral	single	0.001, 0.01, 0.1, 0.3, 1, 2.5, 5, 10, 20, 50, 100, 250, 500	✓												✓	
Hwang2022a [45]	PKDB00923	dap	oral	single	10	✓					✓			✓					
Imamura2013 [46]	PKDB00893	dap	oral	single	10					✓	✓								
Jang2020 [47]	PKDB00913	dap	oral	multi	10	✓					✓								
Kasichayanula2011 [9]	PKDB00841	dap	oral	single	10	✓		✓			✓			✓					
Kasichayanula2011a [48]	PKDB00842	dap	oral	single, multi	2.5, 10, 20, 50	✓				✓	✓			✓	✓			✓	
Kasichayanula2011b [13]	PKDB00843	dap	oral	single	10	✓					✓			✓		✓	✓		
Kasichayanula2011c [49]	PKDB00924	dap	oral	single	20, 50	✓					✓								
Kasichayanula2012 [50]	PKDB00925	dap	oral	single	20	✓					✓								
Kasichayanula2013 [11]	PKDB00844	dap	oral	single, multi	20, 50	✓	✓			✓	✓	✓		✓	✓		✓		
Kasichayanula2013a [51]	PKDB00845	dap	oral	single	10	✓					✓	✓		✓			✓	✓	
Khomitskaya2018 [52]	PKDB00926	dap	oral	single	10	✓					✓								
Kim2023 [53]	PKDB00927	dap	oral	multi	10	✓					✓								
Kim2023a [54]	PKDB00928	dap	oral	single	10	✓					✓								
Komoroski2009 [14]	PKDB00846	dap	oral	single, multi	2.5, 10, 20, 50, 100, 250, 500	✓					✓	✓			✓	✓	✓	✓	
LaCreta2016 [15]	PKDB00847	dap	oral	single	2.5, 10	✓					✓					✓	✓		
Obermeier2010 [5]	PKDB00848	dap, [14C]dap	oral	single	50	✓					✓								
Sha2015 [10]	PKDB00891	dap	oral	single	10	✓					✓						✓	✓	✓
Shah2019a [16]	PKDB00849	dap	oral	single	5	✓					✓					✓	✓		
vanderAartvanderBeek2020 [55]	PKDB00929	dap	oral	single	10	✓					✓								
Watada2019 [56]	PKDB00850	dap	oral	multi	5, 10				✓		✓			✓				✓	
Yang2013 [57]	PKDB00851	dap	oral	single, multi	5, 10	✓					✓	✓		✓	✓		✓	✓	

## Data Availability

All curated pharmacokinetic and pharmacodynamic data are publicly available in the PK-DB database (https://pk-db.com) with unique study identifiers as referenced in the manuscript (Table 1).

## Supplementary Materials

The following supporting information can be downloaded at: https://www.mdpi.com/article/10.3390/pharmaceutics18030287/s1, Figure S1: PRISMA flow diagram; Figure S2: SBML graph of the intestine model of dapagliflozin; Figure S3: SBML graph of the liver model of dapagliflozin; Figure S4: SBML graph of the kidney model of dapagliflozin; Figure S5: Optimization Performance; Figure S6: Simulation Boulton2013; Figure S7: Simulation Cho2021; Figure S8: Simulation FDAMB102006; Figure S9: Simulation Hwang2022a; Figure S10: Simulation Imamura2013; Figure S11: Simulation Jang2020; Figure S12: Simulation Kasichayanula2011c; Figure S13: Simulation Kasichayanula2012; Figure S14: Simulation Kasichayanula2013a; Figure S15: Simulation Khomitskaya2018; Figure S16: Simulation Kim2023; Figure S17: Simulation Kim2023a; Figure S18: Simulation Obermeier2010; Figure S19: Simulation Sha2015; Figure S20: Simulation vanderAartvanderBeek2020; Figure S21: Effect of plasma glucose on pharmacokinetics and pharmacodynamics of dapagliflozin; Figure S22: Local sensitivity analysis; Table S1: Summary of published computational models for dapagliflozin; Table S2: Optimized parameters for the dapagliflozin pharmacokinetic model; Table S3: Optimized parameters for the dapagliflozin pharmacodynamic model.
