# Supplementary material for: A Physiologically Based Pharmacokinetic and Pharmacodynamic (PBPK/PD) Model of Dapagliflozin in Type 2 Diabetes Mellitus: The Effect of Dosing, Hepatorenal Impairment, and Food
Source: Pharmaceutics. 2026 Feb 26;18(3):287. doi: 10.3390/pharmaceutics18030287 (PMC13028959; doi:10.3390/pharmaceutics18030287)
Supplement: Supplementary file 1 [file pharmaceutics-18-00287-s001.zip › pharmaceutics-4014872-supplementary.pdf]

pharmaceutics

Article

A Physiologically Based Pharmacokinetic and Pharmacodynamic (PBPK/PD) Model of Dapagliflozin in Type 2 Diabetes Mellitus: The Effect of Dosing, Hepatorenal Impairment, and Food

Nike Nemitz [1], Michelle Elias [1] and Matthias König [1,2,*]

[1] Faculty of Life Science, Institute for Biology, Systems Medicine of the Liver, Humboldt-Universität zu Berlin, Unter den Linden 6, 10099 Berlin, Germany; nike.philine.elise@gmail.com (N.N.); eliasmic@hu-berlin.de (M.E.)
[2] Institute of Structural Mechanics and Dynamics in Aerospace Engineering, University of Stuttgart, Pfaffenwaldring 27, 70569 Stuttgart, Germany
* Correspondence: koenigmx@hu-berlin.de

Contents

S1. Published Dapagliflozin Models (Tab. S1) 2

S2. Prisma Workflow (Fig. S1) 4

S3. Submodel Visualizations (Fig. S2–S4) 4

S4. Model Equations 5
- S4.1. Intestine Model 5
- S4.2. Liver Model 6
- S4.3. Kidney Model 6
- S4.4. Pharmacodynamics Model 7

S5. Parameter Optimization 8
- S5.1. Optimal Parameters (Tab. S2– S3) 8
- S5.2. Parameter Optimization Results (Fig. S22) 9

S6. Simulations 9
- S6.1. Boulton2013 (Fig. S6) 9
- S6.2. Cho2021 (Fig. S7) 10
- S6.3. FDAMB102006 (Fig. S8) 10
- S6.4. Hwang2022a (Fig. S9) 10
- S6.5. Imamura2013 (Fig. S10) 11
- S6.6. Jang2020 (Fig. S11) 11
- S6.7. Kasichayanula2011c (Fig. S12) 11
- S6.8. Kasichayanula2012 (Fig. S13) 12
- S6.9. Kasichayanula2013a (Fig. S14) 12
- S6.10. Khomitskaya2018 (Fig. S15) 12
- S6.11. Kim2023 (Fig. S16) 13
- S6.12. Kim2023a (Fig. S17) 13
- S6.13. Obermeier2010 (Fig. S18) 13
- S6.14. Sha2015 (Fig. S19) 14
- S6.15. vanderAartvanderBeek2020 (Fig. S20) 14
- S6.16. Glucose Dependency (Fig. S21) 14

S7. Sensitivity Analysis 15

check for updates

Academic Editor: Antonello Di Paolo

Received: 13 November 2025
Revised: 21 December 2025
Accepted: 18 February 2026
Published: 24 February 2026

Copyright: © 2026 by the authors. Licensee MDPI, Basel, Switzerland. This article is an open access article distributed under the terms and conditions of the Creative Commons Attribution (CC BY) license.

S1. Published Dapagliflozin Models (Tab. S1)

Table S1. **Summary of published computational models for dapagliflozin.** Overview of published computational models including model type, software/platform, reproducibility criteria (open software, open model, open code, open data, reproducibility, FAIR, longterm storage), resources, clinical data sources, and model scope.

Study	PubMed ID	Model Type	Platform/Software	Open Software	Open Model	Open Code	Open Data	Reproduci-bility	FAIR	Longterm Storage	Resources	Studies	Clinical Data Used	Scope
Balazki2018 [?]	30270578	QSP + PBPK/PD	PK-Sim, MoBi, OSPS, R	Yes	Yes	Yes	Yes	No	No	No	GitHub repository, Model, Data, Code in supplement; GPL-2.0 license	7	7 clinical studies; digitized literature data	Renal hyperfiltration, tubuloglomerular feedback, glucose-sodium reabsorption, SGLT2 inhibition effects on GFR
Busse2019 [?]	31077437	PopPK + PK/PD	NONMEM, PsN, R	No	No	No	No	No	No	No	-	2	2 clinical studies (T1DM adolescents + adults; PK + UGE ER); clinical studies referenced	Pharmacokinetics, exposure-urinary glucose excretion relationship, body weight and eGFR effects in Type 1 diabetes (adolescents vs adults)
Callegari2021 [?]	33314761	PBPK	Simcyp	No	No	No	No	No	No	No	-	1	1 clinical DDI study (ertugliflozin + mefenamic acid), literature PK for dapagliflozin;	PBPK modeling (ertugliflozin), UGT-mediated drug-drug interaction prediction with mefenamic acid, absorption and metabolism pathways
Guo2025 [?]	40230691	PBPK/PD	PK-Sim, MoBi, OriginLab	Yes	No	No	No	No	No	No	-	25	25 clinical PK/PD studies	PBPK/PD modeling of four SGLT2 inhibitors (dapagliflozin, canagliflozin, empagliflozin, ipragliflozin), enal tubule structure, renal glucose reabsorption, urinary glucose excretion, dose optimization in T2DM with renal insufficiency
Jo2021 [?]	33439535	PBPK	Simcyp	No	No	No	No	No	No	No	-	7	7 clinical studies; trials registered on ClinicalTrials.gov	PBPK model incorporating UGT1A9 ontogeny for pediatric dose selection, DDIs with rifampin/mefenamic acid, hepatic/renal impairment predictions
Maurer2011 [?]	21870203	PK/PD	NONMEM	No	No	No	No	No	No	No	-	1	1 published study: Komoroski et al. 2009; digitized literature data; data not shared	Biologically-based PK/PD model of UGE, rat-to-human translational pharmacology
Mori2016 [?]	27604638	PBPK/PD	Simcyp, simBio	No	No	No	No	No	No	No	-	14	14 studies total: 8 canagliflozin studies, 6 dapagliflozin studies; clinical studies referenced	PBPK/PD model predicting canagliflozin and dapagliflozin concentrations in intestinal lumen and renal proximal tubules, SGLT1/2 inhibition ratios, urinary glucose excretion validation
Shah2021 [?]	33368935	QSP PK/PD	Monolix, R	No	No	No	No	No	No	No	-	5	5 studies total (1 phase IIa + 4 phase III), trials registered on ClinicalTrials.gov	Quantitative systems pharmacology (QSP) model integrating dapagliflozin PK, glucose-insulin homeostasis, renal glucose reabsorption, and HbA1c formation to predict treatment effect in T2DM patients
Shahidehpour2024 [?]	39160349	Mechanistic PK	Python	Yes	No	No	No	No	No	No	-	-	Secondary literature values (number of studies not specified)	Methodology for estimating drug clearance in chronic kidney disease (CKD) using probability density functions from secondary data, mechanistic models, and PK first principles applied to metformin and dapagliflozin PK modeling and dose adjustment
Sokolov2019 [?]	30456904	PK/PD	NONMEM, R	No	No	No	No	No	No	No	-	2	2 clinical studies (not publicly available)	Exposure-response modeling of dapagliflozin PK and 24h-UGE in T1DM patients, comparing Japanese vs non-Japanese populations with covariate effects (eGFR, SMBG, insulin dose)
vanderAart-vanderBeek2021 [?]	33587286	PopPK	NONMEM, R	No	No	No	No	No	No	No	-	1	1 clinical trial	Population PK modeling in non-diabetic CKD patients and linking exposure to changes in kidney risk markers
VanDerWalt2013 [?]	23887724	PopPK	NONMEM, R	No	Yes	Yes	No	No	No	No	NONMEM code in supplement	3	3 clinical studies	Population PK model for dapagliflozin and its inactive metabolite D3OG, quantifying renal vs hepatic contributions to UGT1A9-mediated metabolism in subjects with renal/hepatic impairment
Yao2023 [?]	36890732	Meta-analytic PK/PD	NONMEM, R	No	No	No	No	No	No	No	-	80	80 papers across 3 drugs; digitized literature data; data not shared	Study-level meta-analysis of PK/PD relationships across SGLT2 inhibitors
Nemitz2025	-	PBPK/PD	SBML, Python	Yes	Yes	Yes	Yes	Yes	Yes	Yes	Model, Data, Code in GitHub repository; CI/CD workflow for reproducibility; long-term storage on Zenodo; Licensed under MIT and CC-BY	28	28 clinical studies; digitized literature data	Whole-body mechanistic PBPK/PD model of dapagliflozin linking ADME to SGLT2-mediated urinary glucose excretion and renal threshold for glucose, capturing dose dependency, renal and hepatic impairment, and food effects, implemented in SBML with full FAIR compliance

S2. Prisma Workflow (Fig. S1)

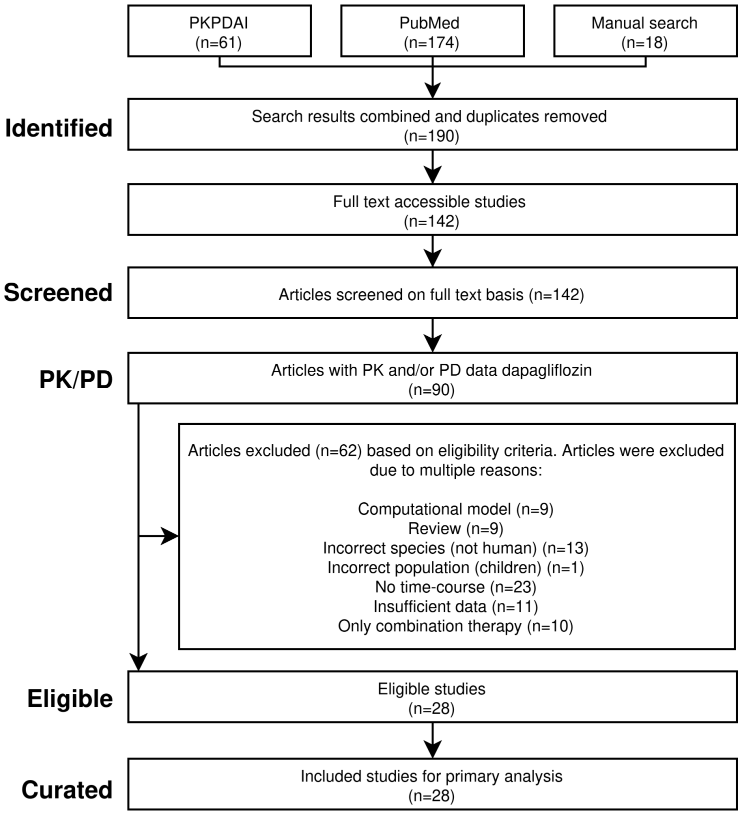

Figure S1. PRISMA flow diagram. Overview of the literature search and data selection process used to establish the dapagliflozin dataset in this work. Studies were identified through PubMed, PKPDAI, and manual searches. Application of the predefined eligibility criteria resulted in 28 included studies. The complete Zotero literature library is publicly available at https://www.zotero.org/groups/6355063/dapagliflozin-model/library.

S3. Submodel Visualizations (Fig. S2–S4)

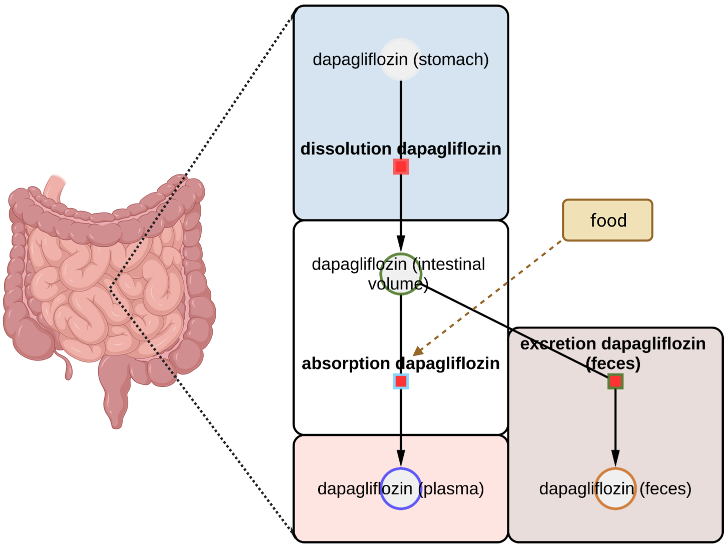

Figure S2. SBML graph of the intestine model of dapagliflozin. After oral administration, dapagliflozin is dissolved in the stomach and enters the intestine. A large fraction of the dose (84%) is absorbed and enters the plasma, the remainder (16%) is excreted in the feces. Food intake can change the absorption rate in the model.

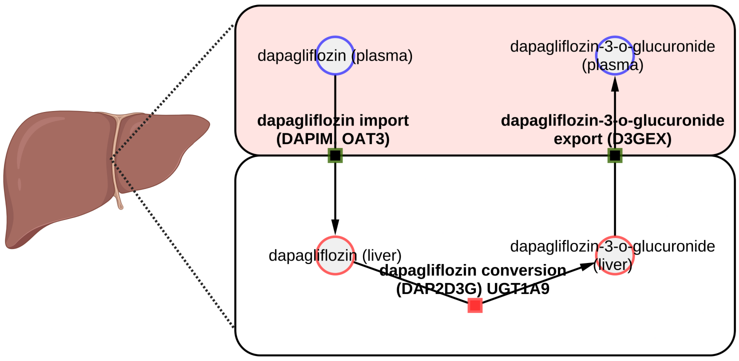

Figure S3. SBML graph of the liver model of dapagliflozin. Dapagliflozin in the plasma of the liver is imported into hepatic tissue via organic anion transporter 3 (OAT3). Within the liver, it undergoes conversion to D3G via UGT1A9. The resulting D3G is then exported into plasma.

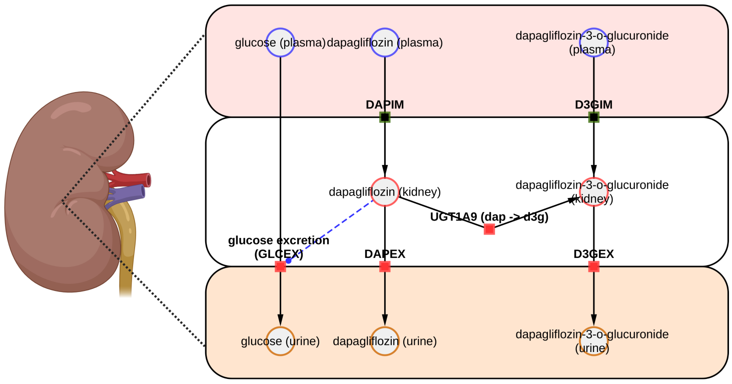

Figure S4. SBML graph of the kidney model of dapagliflozin. Plasma glucose is filtered in the kidney and partly reabsorbed, with the remaining fraction excreted into the urine. Dapagliflozin is imported into renal tissue, where a portion is excreted unchanged into the urine. In the kidney, dapagliflozin is also metabolized to D3G via UGT1A9. D3G in plasma is imported into renal tissue and subsequently excreted in the urine.

S4. Model Equations

The complete model, including simulation scripts and documentation, is available in SBML format under a CC-BY 4.0 license via GitHub (https://github.com/matthiaskoenig/dapagliflozin-model) and archived on Zenodo (model version 0.9.8) [?].

S4.1. Intestine Model

The dissolution process, which determines how quickly the drug enters the intestine, is described via:

$$\text{dissolution}_{\text{dap}} = \frac{Ka_{\text{dis,dap}}}{60\ \frac{min}{hr}} \cdot \frac{\text{PODOSE}_{\text{dap}}}{M_r^{\text{dap}}}$$

Once dissolved, the drug is absorbed from the gut lumen at a rate determined by the absorption scaling factor $f_{\text{absorption}}$ (modified by food), the absorption rate constant DAPABS_{k}, the intestinal volume V_{gu}, and the local drug concentration dap:

$$\text{DAPABS} = f_{\text{absorption}} \cdot \text{DAPABS}_k \cdot V_{\text{gu}} \cdot \text{dap}_{\text{lumen}} \quad [\text{mmol/min}]$$

Only a fraction of the drug $F_{\text{dap,abs}}$ is absorbed and reaches the portal vein:

$$\text{absorption} = F_{\text{dap,abs}} \cdot \text{absorption}$$

The remainder is excreted via the feces:

$$\mathrm{DAPABS} = (1 - F_{\mathrm{dap,abs}}) \cdot \mathrm{absorption}$$

The resulting differential equations are:

$$\frac{d\,\mathrm{PODOSE_{dap}}}{dt} = -\mathrm{dissolution_{dap}} \cdot M_r^{\mathrm{dap}}$$

$$\frac{d\,\mathrm{dap_{ext}}}{dt} = \frac{\mathrm{DAPABS}}{V_{\mathrm{ext}}}$$

$$\frac{d\,\mathrm{dap_{feces}}}{dt} = \mathrm{DAPEXC}$$

$$\frac{d\,\mathrm{dap_{lumen}}}{dt} = -\frac{\mathrm{DAPABS}}{V_{\mathrm{lumen}}} - \frac{\mathrm{DAPEXC}}{V_{\mathrm{lumen}}} + \frac{\mathrm{dissolution_{dap}}}{V_{\mathrm{lumen}}}$$

S4.2. Liver Model

Dapagliflozin is taken up into the liver (DAPIM) via transport proteins, possibly OAT3, and represented by reversible Michaelis–Menten kinetics with $K_m = 33, \mu$M [**?**].

$$\mathrm{DAPIM} = \frac{\mathrm{DAPIM_{Vmax}}}{\mathrm{DAPIM_{Km,dap}}} \cdot V_{\mathrm{li}} \cdot \frac{\mathrm{dap_{ext}} - \mathrm{dap}}{1 + \frac{\mathrm{dap_{ext}}}{\mathrm{DAPIM_{Km,dap}}} + \frac{\mathrm{dap}}{\mathrm{DAPIM_{Km,dap}}}}$$

The conversion of dapagliflozin to dapagliflozin-3-O-glucuronide is catalyzed by uridine diphosphate glucuronosyltransferase 1A9 (UGT1A9) and modeled as irreversible Michaelis–Menten kinetics.

$$\mathrm{DAP2D3G} = f_{\mathrm{ugt1a9}} \cdot \mathrm{DAP2D3G_{Vmax}} \cdot V_{\mathrm{li}} \cdot \frac{\mathrm{dap}}{\mathrm{dap} + \mathrm{DAP2D3G_{Km,dap}}}$$

After conversion to dapagliflozin-3-O-glucuronide, the metabolite is exported, possibly via OAT3 ($K_m = 115, \mu$M [**?**]), and modeled as reversible Michaelis–Menten kinetics.

$$\mathrm{D3GEX} = \frac{\mathrm{D3GEX_{Vmax}}}{\mathrm{D3GEX_{Km,d3g}}} \cdot V_{\mathrm{li}} \cdot \frac{\mathrm{d3g} - \mathrm{d3g_{ext}}}{1 + \frac{\mathrm{d3g}}{\mathrm{D3GEX_{Km,d3g}}} + \frac{\mathrm{d3g_{ext}}}{\mathrm{D3GEX_{Km,d3g}}}}$$

The resulting differential equations are:

$$\frac{d\,\mathrm{d3g}}{dt} = \frac{\mathrm{DAP2D3G}}{V_{\mathrm{li}}} - \frac{\mathrm{D3GEX}}{V_{\mathrm{li}}}$$

$$\frac{d\,\mathrm{d3g_{ext}}}{dt} = \frac{\mathrm{D3GEX}}{V_{\mathrm{ext}}}$$

$$\frac{d\,\mathrm{dap}}{dt} = \frac{\mathrm{DAPIM}}{V_{\mathrm{li}}} - \frac{\mathrm{DAP2D3G}}{V_{\mathrm{li}}}$$

$$\frac{d\,\mathrm{dap_{ext}}}{dt} = -\frac{\mathrm{DAPIM}}{V_{\mathrm{ext}}}$$

S4.3. Kidney Model

Dapagliflozin enters renal tissue (DAPIM), where a fraction is excreted unchanged (DAPEX) and the remainder metabolized to dapagliflozin-3-O-glucuronide, which is subsequently excreted in urine (D3GEX). Renal uptake was modeled as carrier-mediated transport, described by reversible Michaelis–Menten kinetics:

$$\mathrm{DAPIM} = \frac{\mathrm{DAPIM_{Vmax}}}{\mathrm{DAPIM_{Km,dap}}} \cdot V_{\mathrm{ki}} \cdot \frac{\mathrm{dap_{ext}} - \mathrm{dap}}{1 + \frac{\mathrm{dap_{ext}}}{\mathrm{DAPIM_{Km,dap}}} + \frac{\mathrm{dap}}{\mathrm{DAPIM_{Km,dap}}}}$$

Similarly, the import of its primary metabolite, dapagliflozin-3-O-glucuronide, into kidney cells is described by the equation:

$$\mathrm{D3GIM} = \frac{\mathrm{D3GIM_{Vmax}}}{\mathrm{D3GIM_{Km,d3g}}} \cdot V_{\mathrm{ki}} \cdot \frac{\mathrm{d3g_{ext}} - \mathrm{d3g}}{1 + \frac{\mathrm{d3g_{ext}}}{\mathrm{D3GIM_{Km,d3g}}} + \frac{\mathrm{d3g}}{\mathrm{D3GIM_{Km,d3g}}}}$$

Within the kidney, dapagliflozin is metabolized to dapagliflozin-3-O-glucuronide by UGT1A9 (process DAP2D3G), modeled as irreversible Michaelis–Menten kinetics:

$$\mathrm{DAP2D3G} = f_{\mathrm{ugt1a9}} \cdot \mathrm{DAP2D3G_{Vmax}} \cdot V_{\mathrm{ki}} \cdot \frac{\mathrm{dap}}{\mathrm{dap} + \mathrm{DAP2D3G_{Km,dap}}}$$

The transport of dapagliflozin and dapagliflozin-3-O-glucuronide were assumed to be fast compared to the metabolic conversion.

Both dapagliflozin and dapagliflozin-3-O-glucuronide are excreted via the kidney in the urine. Their renal excretion rates are given by:

$$\mathrm{DAPEX} = f_{\mathrm{renal\ function}} \cdot \mathrm{D3GEX}_k \cdot V_{\mathrm{ki}} \cdot \mathrm{d3g_{ext}}$$

$$\mathrm{D3GEX} = f_{\mathrm{renal\ function}} \cdot \mathrm{DAPEX}_k \cdot V_{\mathrm{ki}} \cdot \mathrm{dap_{ext}}$$

The parameter $\mathrm{f_{renal\_function}}$ was introduced as a scaling factor for renal function.

Kidney volume $\mathrm{V_{ki}}$ was set to 0.44% of bodyweight to scale concentration-dependent processes such as renal transport and metabolism, ensuring physiological relevance.

The resulting differential equations are:

$$\frac{d\,\mathrm{d3g}}{dt} = \frac{\mathrm{D3GIM}}{V_{\mathrm{ki}}} + \frac{\mathrm{DAP2D3G}}{V_{\mathrm{ki}}}$$

$$\frac{d\,\mathrm{d3g_{ext}}}{dt} = -\frac{\mathrm{D3GIM}}{V_{\mathrm{ext}}} - \frac{\mathrm{D3GEX}}{V_{\mathrm{ext}}}$$

$$\frac{d\,\mathrm{d3g_{urine}}}{dt} = \mathrm{D3GEX}$$

$$\frac{d\,\mathrm{dap}}{dt} = \frac{\mathrm{DAPIM}}{V_{\mathrm{ki}}} - \frac{\mathrm{DAP2D3G}}{V_{\mathrm{ki}}}$$

$$\frac{d\,\mathrm{dap_{ext}}}{dt} = -\frac{\mathrm{DAPIM}}{V_{\mathrm{ext}}} - \frac{\mathrm{DAPEX}}{V_{\mathrm{ext}}}$$

$$\frac{d\,\mathrm{dap_{urine}}}{dt} = \mathrm{DAPEX}$$

S4.4. Pharmacodynamics Model

The pharmacodynamic model describes how urinary glucose excretion (UGE) is affected by dapagliflozin. UGE depends on the renal threshold for glucose (RTG), defined as the plasma glucose concentration at which renal reabsorption becomes saturated [**?**]. Basal RTG is determined by fasting plasma glucose (FPG) and reduced by dapagliflozin:

$$\mathrm{RTG_{fpg}} = \mathrm{RTG_{base}} + \mathrm{RTG_{m,\,fpg}} \cdot (\mathrm{glc_{ext}} - fpg_{\mathrm{healthy}})$$

$$\mathrm{RTG_{delta}} = \mathrm{RTG_{fpg}} \cdot \mathrm{RTG_{max\ inhibition}}$$

$$\mathrm{RTG} = \mathrm{RTG_{fpg}} - \mathrm{RTG_{delta}} \cdot \frac{\mathrm{dap_{ext}}^{\mathrm{RTG}_\gamma}}{\mathrm{RTG_{E50}}^{\mathrm{RTG}_\gamma} + \mathrm{dap}^{\mathrm{RTG}_\gamma}}$$

Above the RTG, excess glucose that cannot be reabsorbed is excreted in the urine, resulting in increased UGE. Glucose excretion (GLCEX) in this range depends on the glomerular filtration rate (GFR), which governs plasma glucose filtration by the kidneys. Below the RTG, no glucose is excreted.

GFR depends on renal status and is scaled by the parameter $\mathrm{f_{renal\_function}}$, with 1 representing normal and values below 1 reduced renal function.

$$\mathrm{GFR} = f_{\mathrm{renal\_function}} \cdot \mathrm{GFR_{healthy}}$$

$$\mathrm{GLCEX} = \begin{cases} \frac{\mathrm{GFR}}{\mathrm{cf_{ml/l}}} \cdot (\mathrm{glc_{ext}} - \mathrm{RTG}) & \text{if } \mathrm{glc_{ext}} > RTG \\ 0\ \mathrm{mmole/min} & \text{if } \mathrm{glc_{ext}} \leq RTG \end{cases}$$

The plasma glucose concentration $\mathrm{glc_{ext}}$ was assumed to be constant but varied with subject status, with diabetic patients having higher values than healthy subjects.

The change in urinary glucose is determined via the following differential equation:

$$\frac{d\ glc_{urine}}{dt} = GLCEX$$

The cumulative amount of glucose in the urine, i.e., the UGE, is calculated from the amount of glucose in the urine via:

$$\mathrm{UGE} = \frac{\mathrm{glc_{urine}} \cdot M_{\mathrm{rglc}}}{\mathrm{cf_{mg/g}}}$$

S5. Parameter Optimization

S5.1. Optimal Parameters (Tab. S2– S3)

Table S2. Optimized parameters for the dapagliflozin pharmacokinetic model. Lower (LB) and upper (UB) bounds applied during parameter optimization.

Parameter name	Description	Value	Unit	LB	UB
`ftissue_dap`	Tissue blood flow rate for DAP	0.01	1/min	0.01	100
`Kp_dap`	Tissue-to-plasma partition coefficient	25.517	-	1	50
`DAP2D3G_Vmax`	Liver metabolism of DAP to D3G	0.01992	mmol/min/l	0.001	100
`KI__f_DAP2D3G`	Renal DAP2D3G activity relative to liver	10.0	-	0.1	10
`KI__DAPEX_k`	Renal excretion rate of DAP	0.01815	1/min	0.0001	10
`KI__D3GEX_k`	Renal excretion rate of D3G	0.45036	1/min	0.1	10
`GU__Ka_dis_dap`	Dissolution rate of DAP in GI tract	0.84842	1/hr	0.001	100
`GU__DAPABS_k`	Absorption rate of DAP in GI tract	0.05946	1/min	1×10^{-5}	10

Table S3. Optimized parameters for the dapagliflozin pharmacodynamic model. Lower (LB) and upper (UB) bounds applied during parameter optimization.

Parameter name	Description	Value	Unit	LB	UB
`KI__RTG_E50`	Half-maximal effect concentration of DAP on RTG	6.49×10^{-6}	mM	1×10^{-6}	0.1
`KI__RTG_base`	baseline RTG	8.00	mM	8	14
`KI__RTG_gamma`	hill coefficient reduction in RTG	1.036	mM	1	5
`KI__RTG_max_inhibition`	RTG maximum inhibition	0.70673	–	0.2	1.0
`KI__RTG_m_fpg`	FPG effect on RTG	1.2533	–	0.2	3

S5.2. Parameter Optimization Results (Fig. S22)

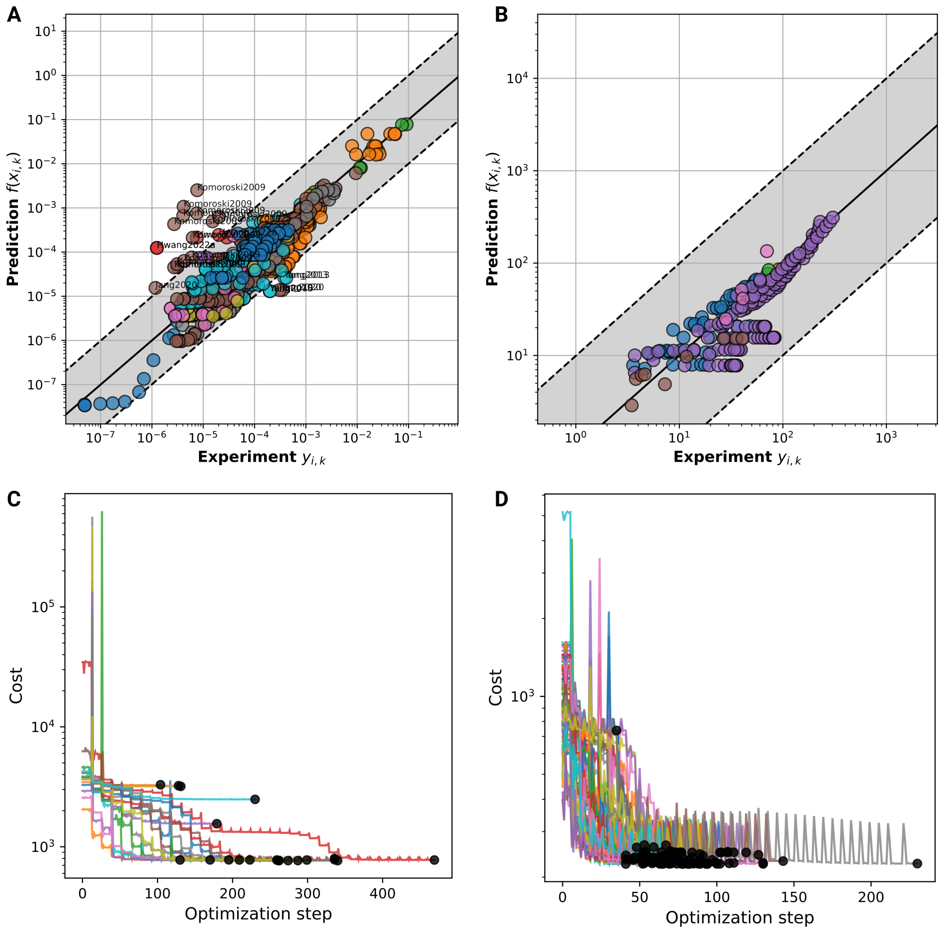

Figure S5. Optimization Performance. Goodness-of-fit (predicted vs. observed intravenous data) and error convergence (cost vs. optimization step) for pharmacokinetic (**A**, **C**) and pharmacodynamic (**B**, **D**) parameter sets.

S6. Simulations

S6.1. Boulton2013 (Fig. S6)

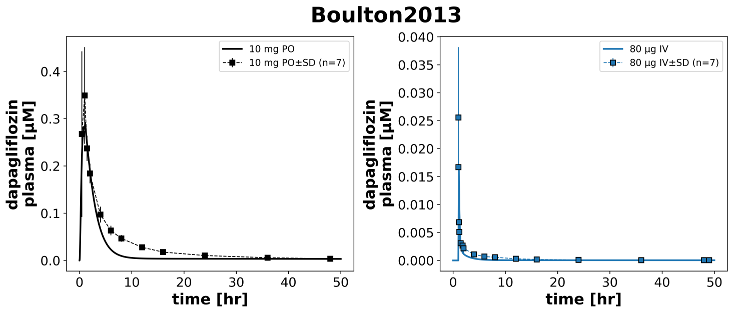

Figure S6. Simulation Boulton2013 [?]. Simulated (solid lines) versus observed (dashed lines with squares) dapagliflozin plasma concentrations after a 10 mg single oral dose or 80 μg IV injection in healthy volunteers.

https://doi.org/10.3390/pharmaceutics1010000

S6.2. Cho2021 (Fig. S7)

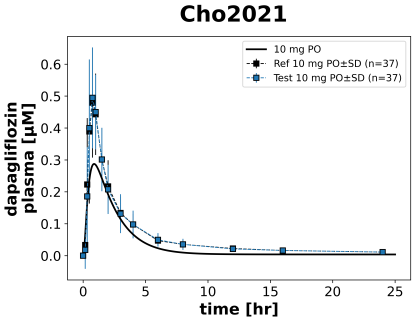

Figure S7. Simulation Cho2021 [?]. Simulated (solid lines) versus observed (dashed lines with squares and SDs) dapagliflozin plasma concentrations after a 10 mg single oral dose (reference or test) in healthy volunteers.

S6.3. FDAMB102006 (Fig. S8)

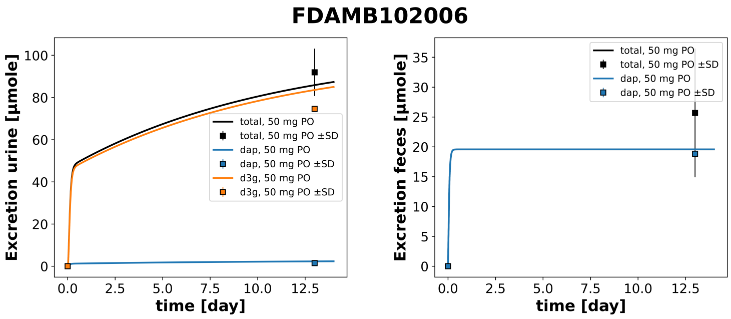

Figure S8. Simulation FDAMB102006 [?]. Simulated (solid lines) versus observed (dashed lines with squares and SDs) dapagliflozin, D3G and total urinary and fecal excretion after a 50 mg single oral dose in healthy volunteers.

S6.4. Hwang2022a (Fig. S9)

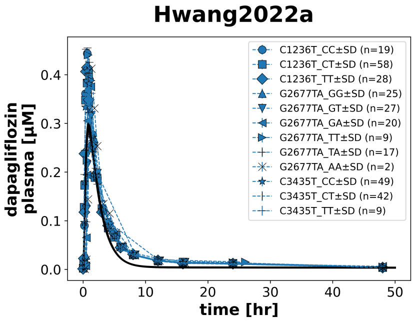

Figure S9. Simulation Hwang2022a [?]. Simulated (solid lines) versus observed (dashed lines with symbols) dapagliflozin plasma concentrations after a 10 mg single oral dose in healthy volunteers with different ABCB1 genotypes.

S6.5. Imamura2013 (Fig. S10)

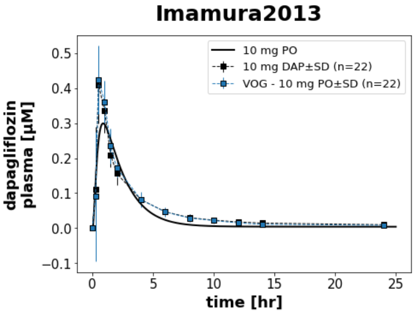

Figure S10. Simulation Imamura2013 [?]. Simulated (solid lines) versus observed (dashed lines with squares and SDs) dapagliflozin plasma concentrations after a 10 mg single oral dose (alone or in combination with voglibose) in healthy volunteers. Error bars represent ±SD (n=22).

S6.6. Jang2020 (Fig. S11)

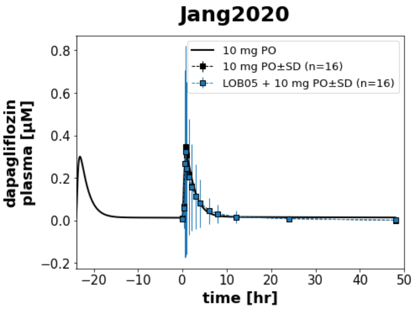

Figure S11. Simulation Jang2020 [?]. Simulated (solid lines) versus observed (dashed lines with squares and SDs) dapagliflozin plasma concentrations after a 10 mg single oral dose (alone, or in combination with lobeglitazone) in healthy volunteers. Error bars represent ±SD (n=16).

S6.7. Kasichayanula2011c (Fig. S12)

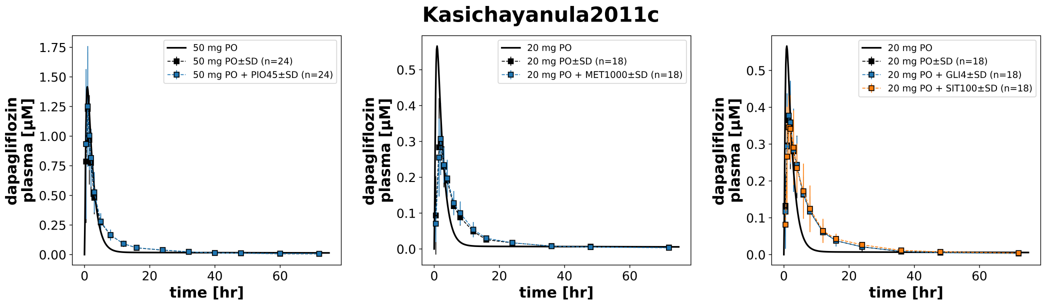

Figure S12. Simulation Kasichayanula2011c [?]. Simulated (solid lines) versus observed (dashed lines with squares and SDs) dapagliflozin plasma concentrations after a 50 mg single oral dose (alone, or in combination with metformin, pioglitazone or glimepiride) in healthy volunteers.

S6.8. Kasichayanula2012 (Fig. S13)

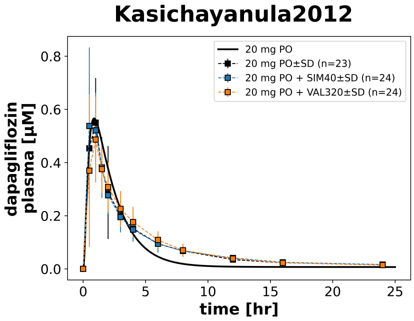

Figure S13. Simulation Kasichayanula2012 [?]. Simulated (solid lines) versus observed (dashed lines with squares and SDs) dapagliflozin plasma concentrations after a 20 mg single oral dose (alone, or in combination with simvastatin or valsartan) in healthy volunteers.

S6.9. Kasichayanula2013a (Fig. S14)

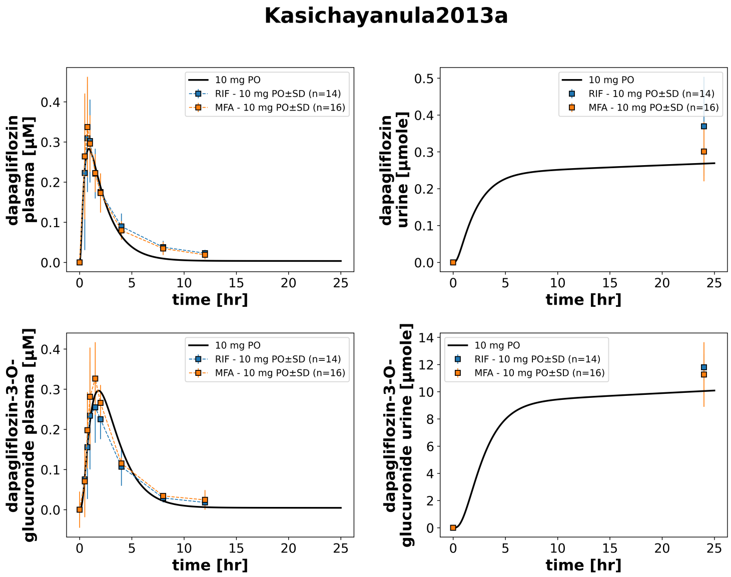

Figure S14. Simulation Kasichayanula2013a [?]. Simulated (solid lines) versus observed (dashed lines with squares and SDs) dapagliflozin and D3G plasma concentrations and fecal excretion after a 10 mg single oral dose (alone, or in combination with rifampin or mefenamic acid) in healthy volunteers.

S6.10. Khomitskaya2018 (Fig. S15)

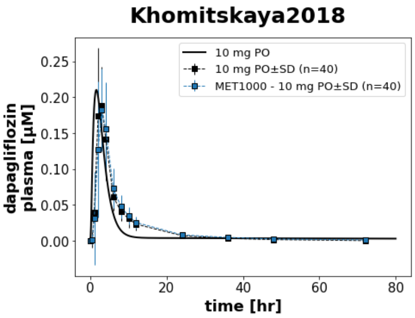

Figure S15. Simulation Khomitskaya2018 [?]. Simulated (solid lines) versus observed (dashed lines with squares and SDs) dapagliflozin plasma concentrations after a 10 mg single oral dose (alone, or in combination with metformin) in healthy volunteers.

S6.11. Kim2023 (Fig. S16)

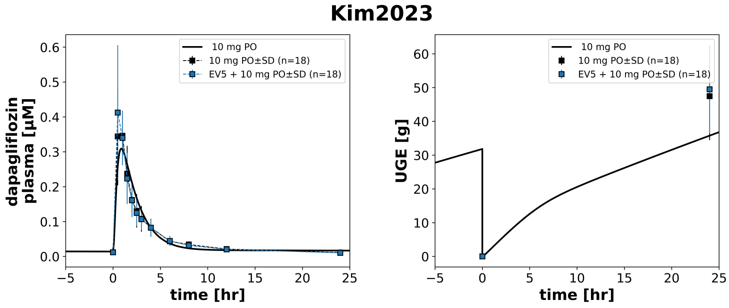

Figure S16. Simulation Kim2023 [?]. Simulated (solid lines) versus observed (dashed lines with squares and SDs) dapagliflozin plasma concentrations after a 10 mg single oral dose (alone, or in combination with evogliptin) in healthy volunteers.

S6.12. Kim2023a (Fig. S17)

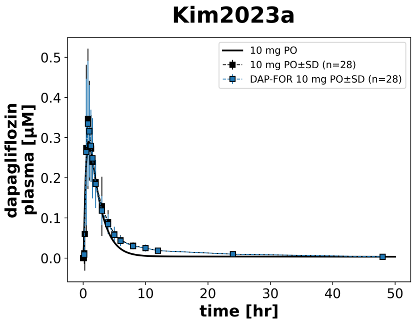

Figure S17. Simulation Kim2023a [?]. Simulated (solid lines) versus observed (dashed lines with squares and SDs) dapagliflozin plasma concentrations after a 10 mg single oral dose (dapagliflozin or dapagliflozin formate) in healthy volunteers.

S6.13. Obermeier2010 (Fig. S18)

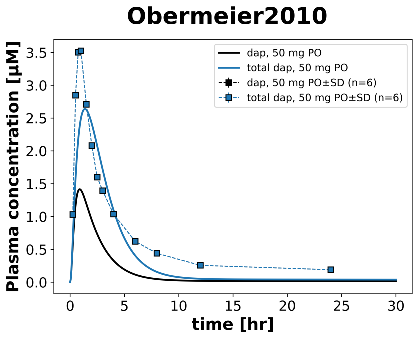

Figure S18. Simulation Obermeier2010 [?]. Simulated (solid lines) versus observed (dashed lines with squares) dapagliflozin plasma concentrations after a 10 mg single oral dose (dapagliflozin or dapagliflozin formate) in healthy volunteers.

S6.14. Sha2015 (Fig. S19)

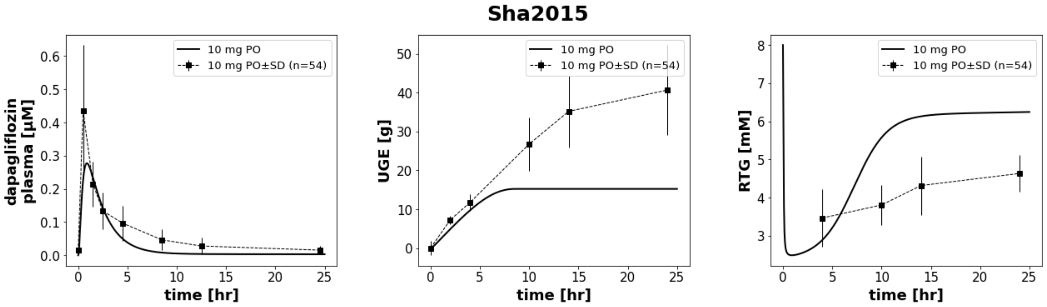

Figure S19. Simulation Sha2015 [?]. Simulated (solid lines) versus observed (dashed lines with squares and SDs) dapagliflozin plasma concentrations, UGE and RTG after a 10 mg single oral dose (dapagliflozin or dapagliflozin formate) in healthy volunteers.

S6.15. vanderAartvanderBeek2020 (Fig. S20)

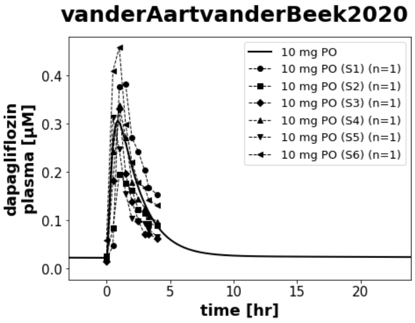

Figure S20. Simulation vanderAartvanderBeek2020 [?]. Simulated (solid lines) versus observed (dashed lines with symbols) dapagliflozin plasma concentrations after a 10 mg single oral dose in healthy volunteers.

S6.16. Glucose Dependency (Fig. S21)

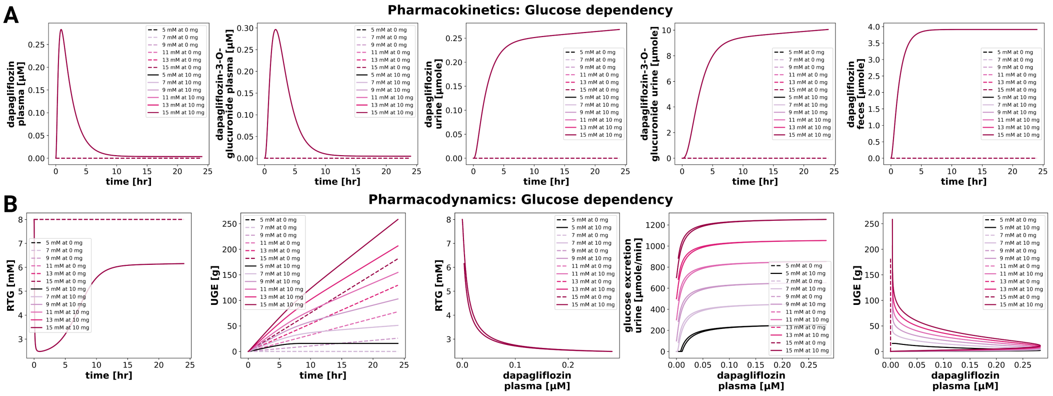

Figure S21. Effect of plasma glucose on pharmacokinetics and pharmacodynamics of dapagliflozin. A) Pharmacokinetic time courses of dapagliflozin and D3G in plasma, urine, and feces at 0 mg or 10 mg dose across plasma glucose concentrations of 3–15 mM. **B)** Pharmacodynamic time courses at 0 mg or 10 mg dose show the effect of plasma glucose (3–15 mM) on RTG and UGE, as well as exposure-response relationships between dapagliflozin plasma concentrations, RTG, glucose excretion, and UGE.

S7. Sensitivity Analysis

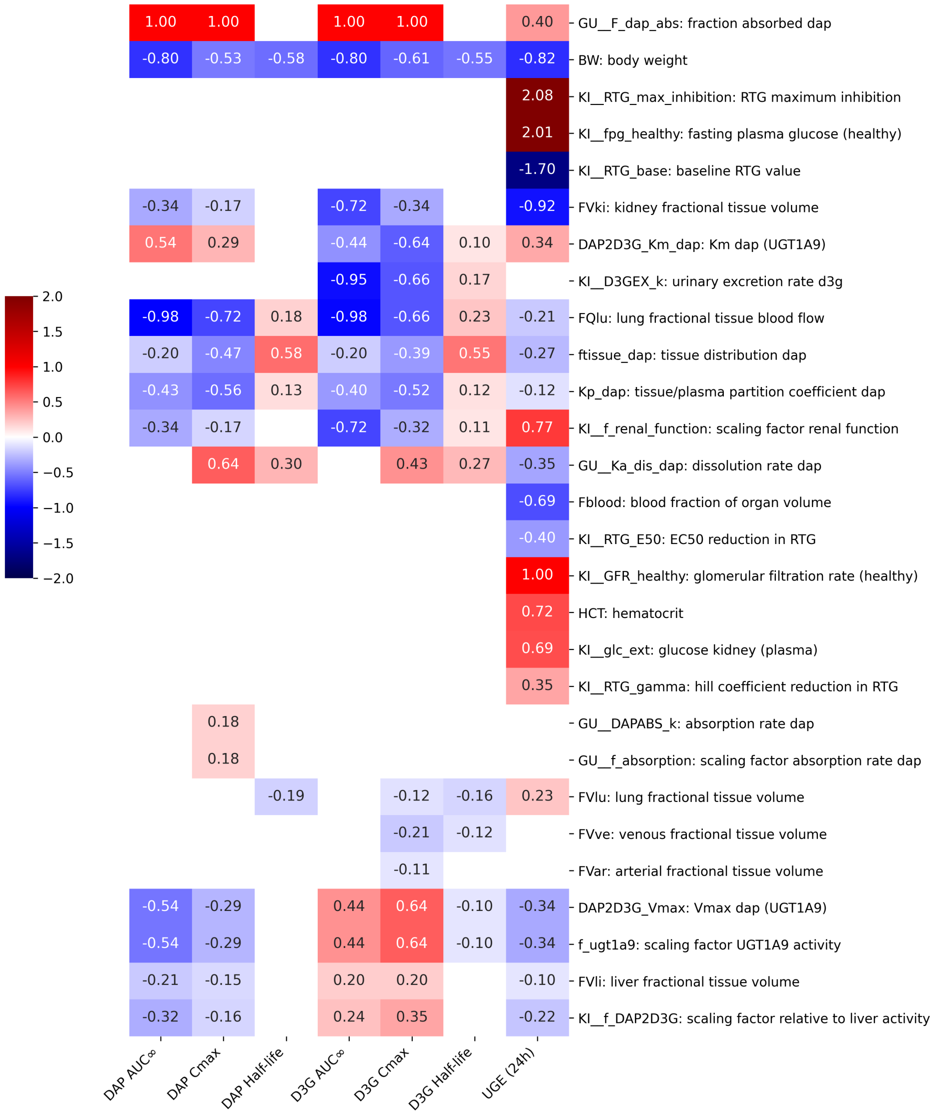

Figure S22. Local sensitivity analysis. Heatmap of normalized local sensitivities showing the effect of individual model parameters on pharmacokinetic and pharmacodynamic readouts. Sensitivities were computed using a symmetric midpoint perturbation with a ±1% change in each parameter and normalized to the parameter change. Blue indicates a positive sensitivity (increase in the output with increasing parameter value), while red indicates a negative sensitivity (decrease in the output). Parameters were hierarchically clustered using the single linkage method. Numerical sensitivity values are annotated only for entries with an absolute value ≥ 0.1; values below this cutoff are shown without text annotations.
