# Supplementary figures and images for: A Physiologically Based Pharmacokinetic and Pharmacodynamic (PBPK/PD) Model of Dapagliflozin in Type 2 Diabetes Mellitus: The Effect of Dosing, Hepatorenal Impairment, and Food
Source: Pharmaceutics. 2026 Feb 26;18(3):287. doi: 10.3390/pharmaceutics18030287 (PMC13028959; doi:10.3390/pharmaceutics18030287)

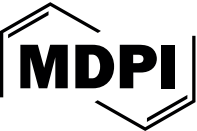

Supplement: Supplementary file 1 [file pharmaceutics-18-00287-s001.zip › Definitions/logo-mdpi-eps-converted-to.pdf]

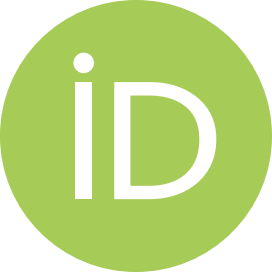

Supplement: Supplementary file 1 [file pharmaceutics-18-00287-s001.zip › Definitions/logo-orcid.pdf]

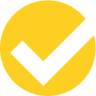

check for  
updates

Supplement: Supplementary file 1 [file pharmaceutics-18-00287-s001.zip › Definitions/logo-updates-eps-converted-to.pdf]

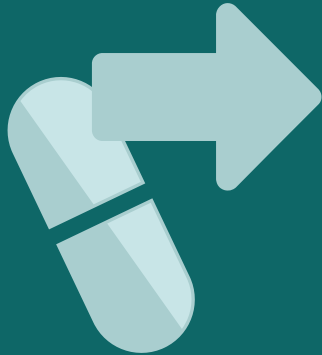

*pharmaceuticals*

Supplement: Supplementary file 1 [file pharmaceutics-18-00287-s001.zip › Definitions/pharmaceutics-logo-eps-converted-to.pdf]

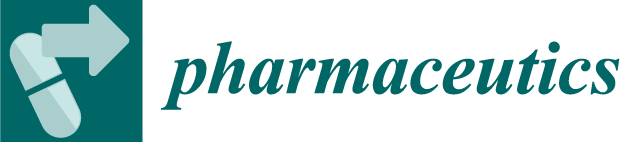

Supplement: Supplementary file 1 [file pharmaceutics-18-00287-s001.zip › Definitions/pharmaceutics-logo.png]

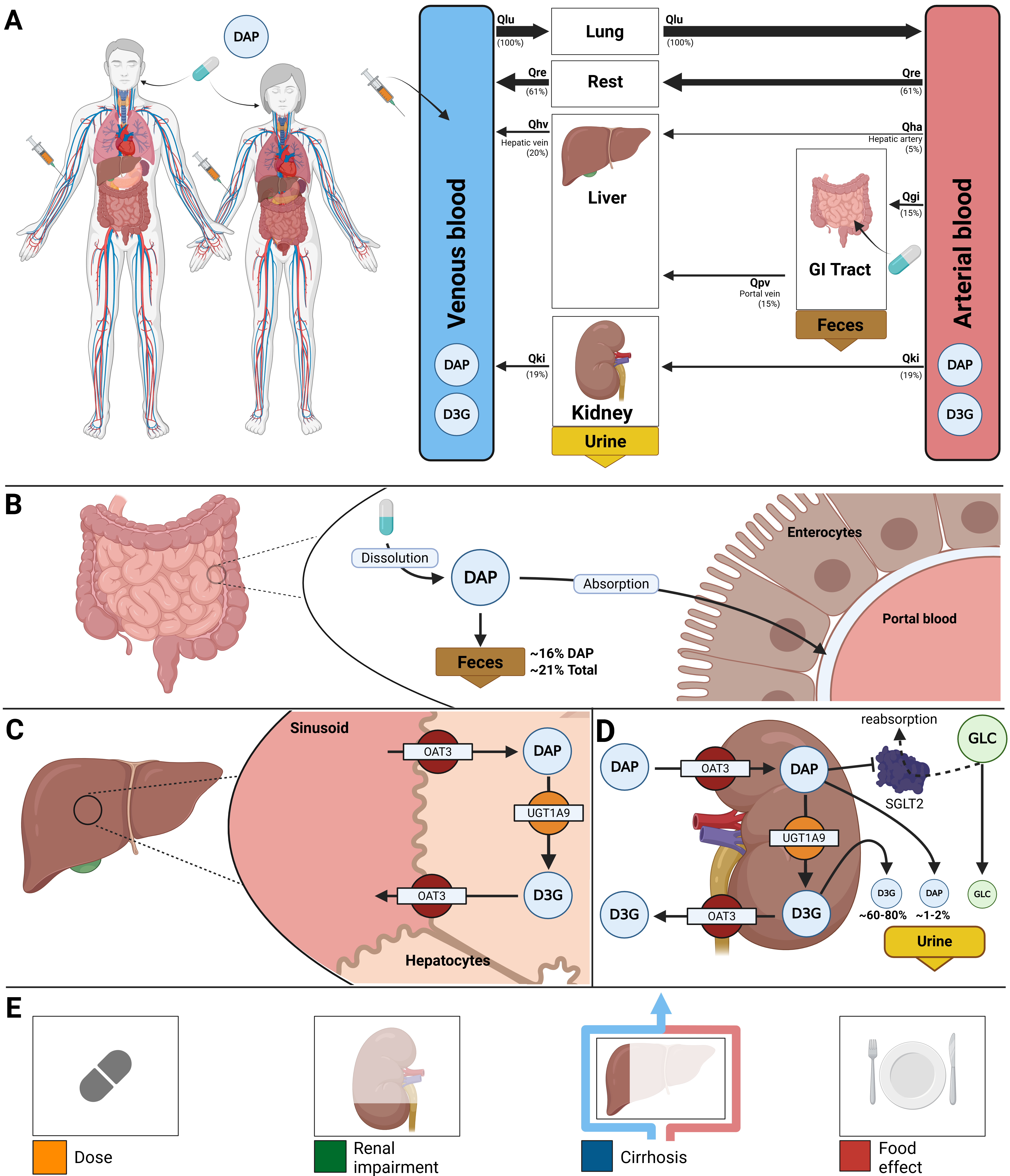

Supplement: Supplementary file 1 [file pharmaceutics-18-00287-s001.zip › Figures/Fig01_dapagliflozin_model.png]

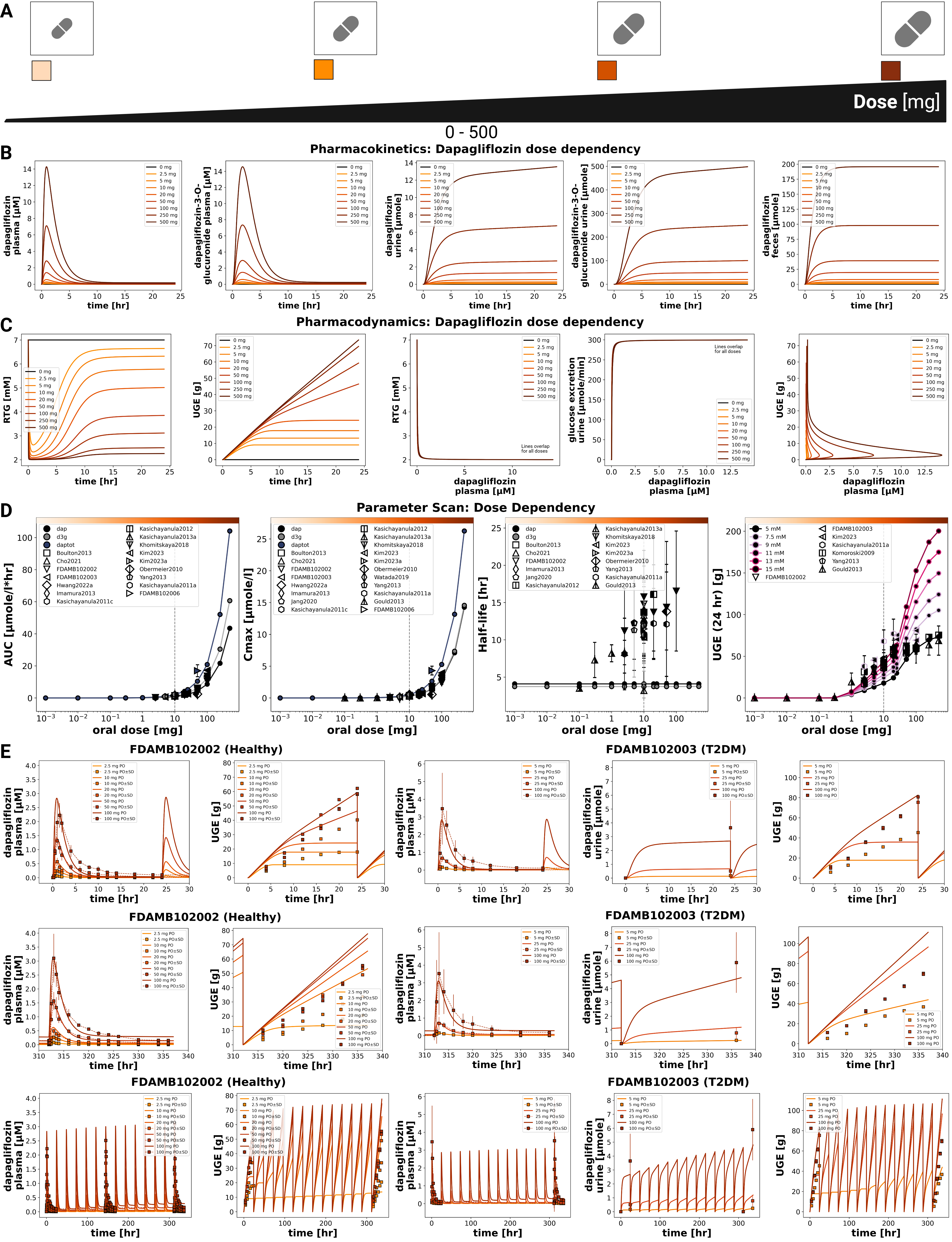

Supplement: Supplementary file 1 [file pharmaceutics-18-00287-s001.zip › Figures/Fig02_dose_dependency_1.png]

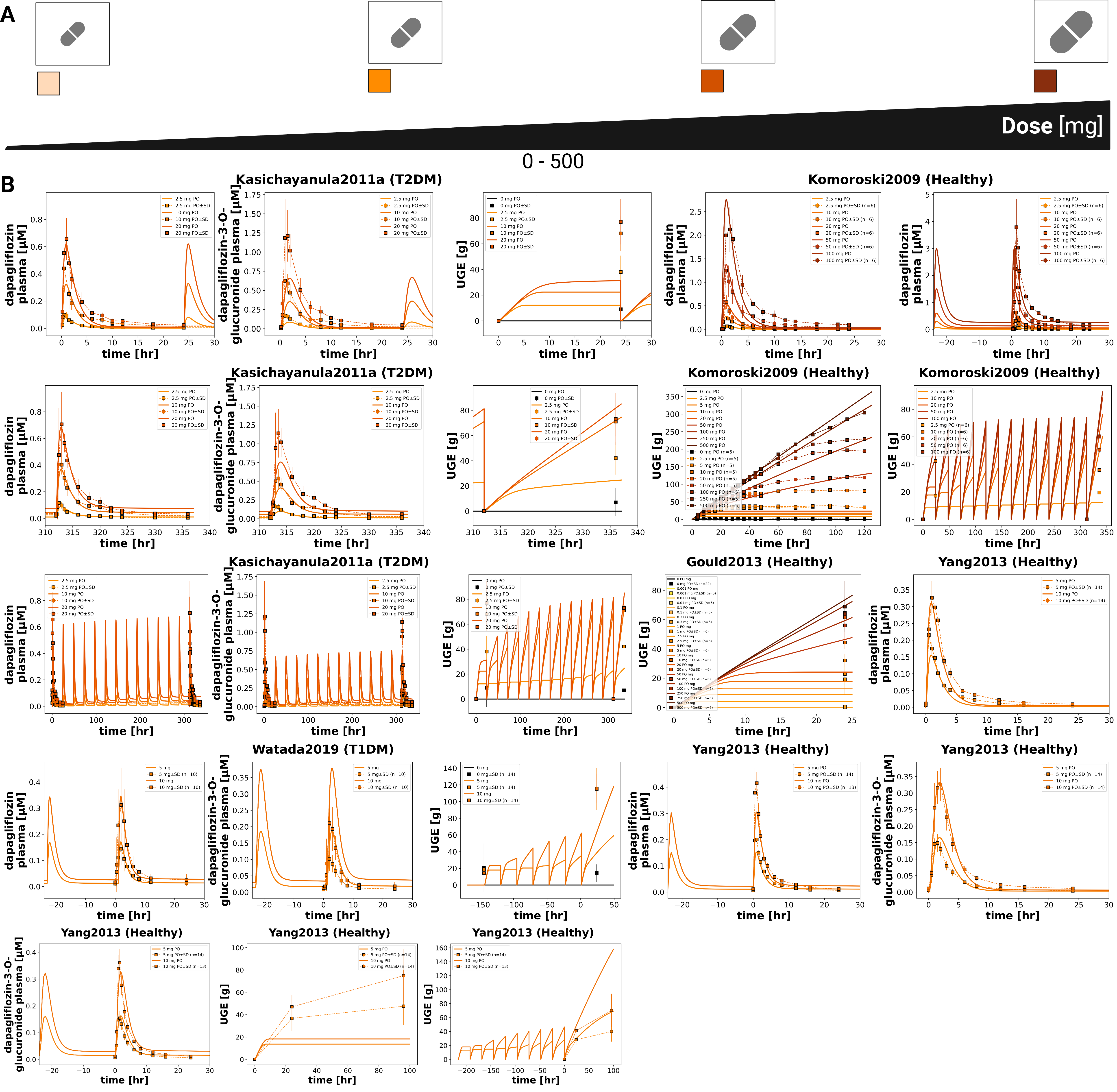

Supplement: Supplementary file 1 [file pharmaceutics-18-00287-s001.zip › Figures/Fig03_dose_dependency_2.png]

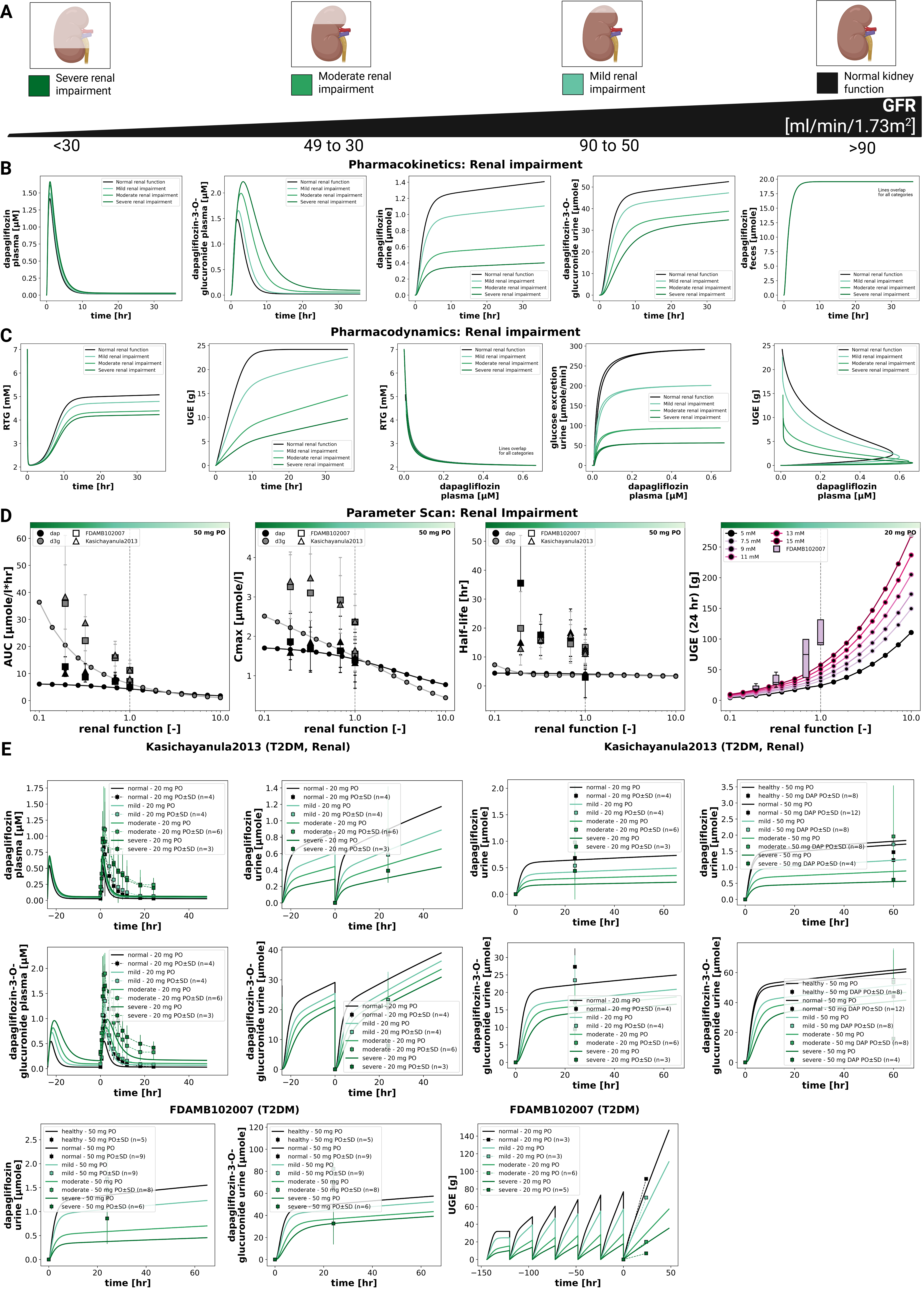

Supplement: Supplementary file 1 [file pharmaceutics-18-00287-s001.zip › Figures/Fig04_renal_impairment.png]

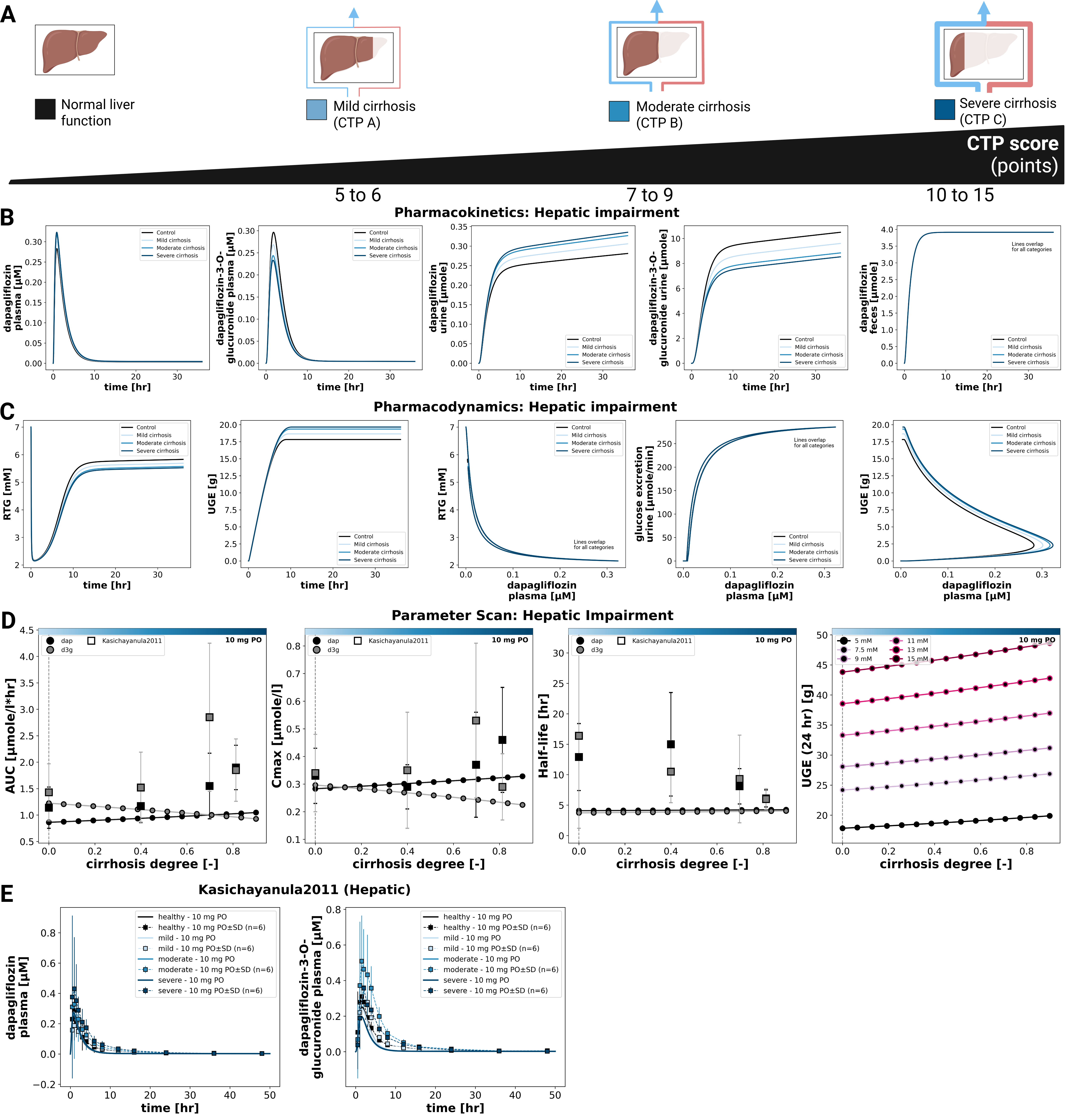

Supplement: Supplementary file 1 [file pharmaceutics-18-00287-s001.zip › Figures/Fig05_hepatic_impairment.png]

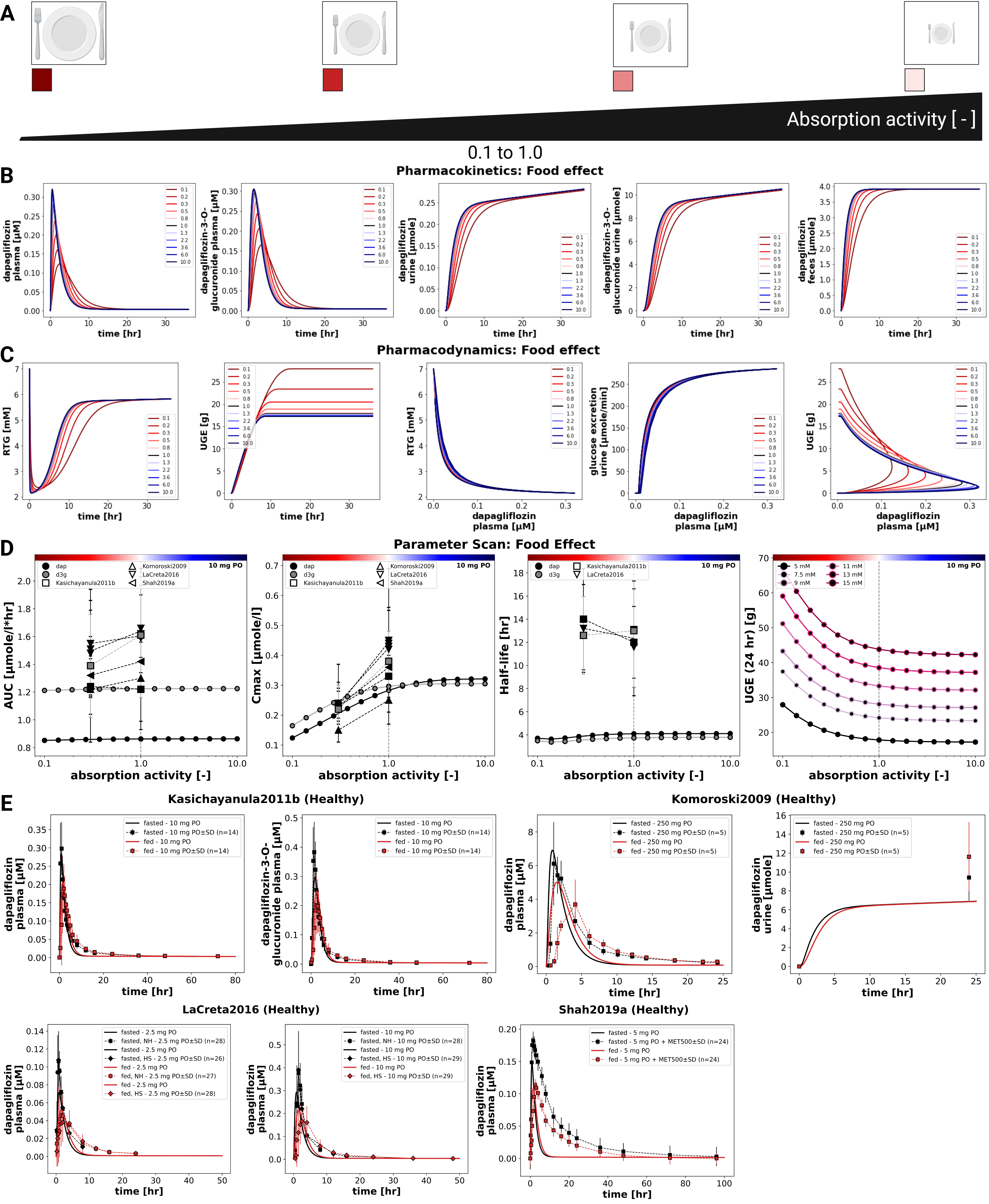

Supplement: Supplementary file 1 [file pharmaceutics-18-00287-s001.zip › Figures/Fig06_food_effect.png]

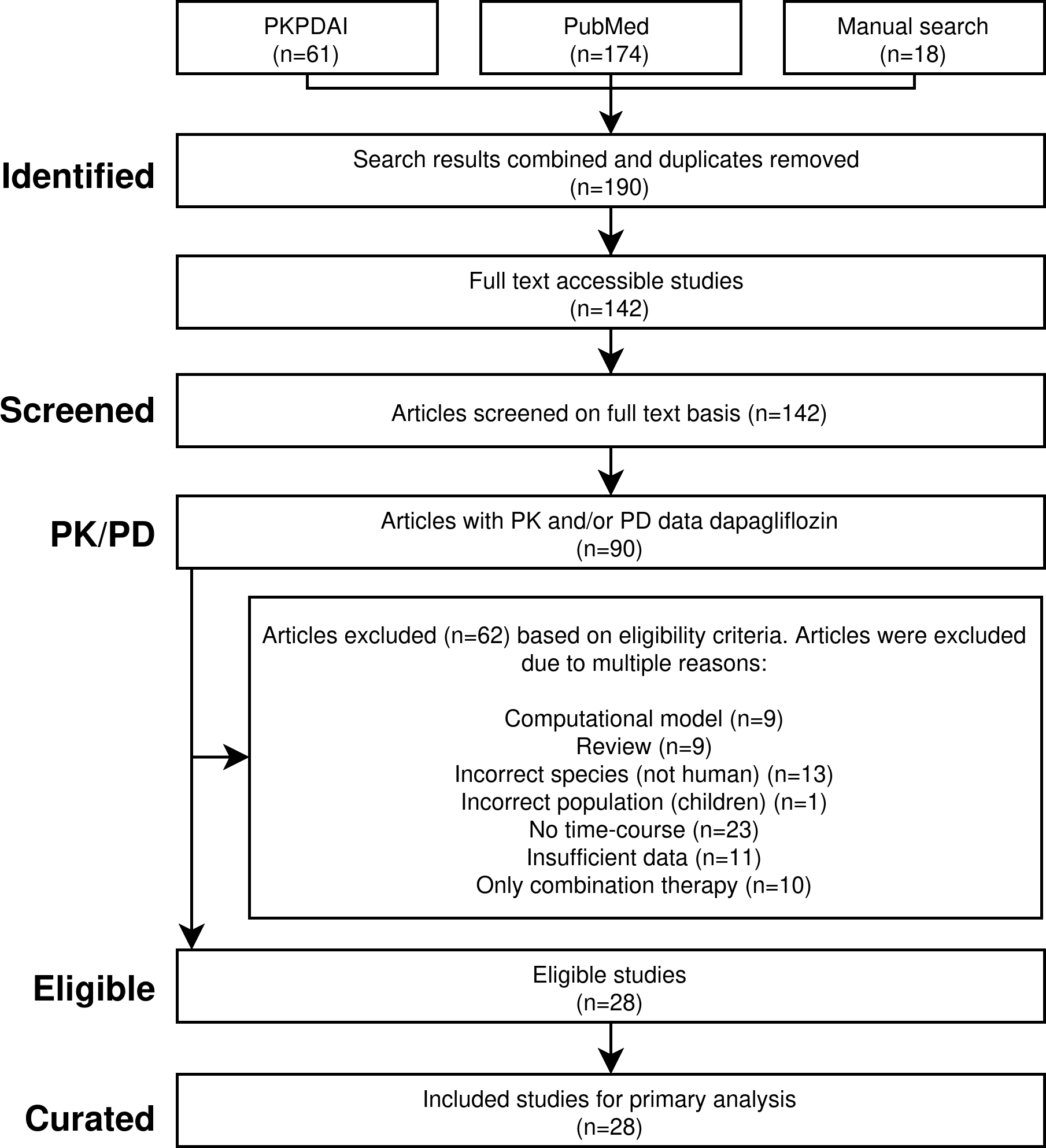

Supplement: Supplementary file 1 [file pharmaceutics-18-00287-s001.zip › Figures/FigS01_dapagliflozin_prisma_diagram.png]

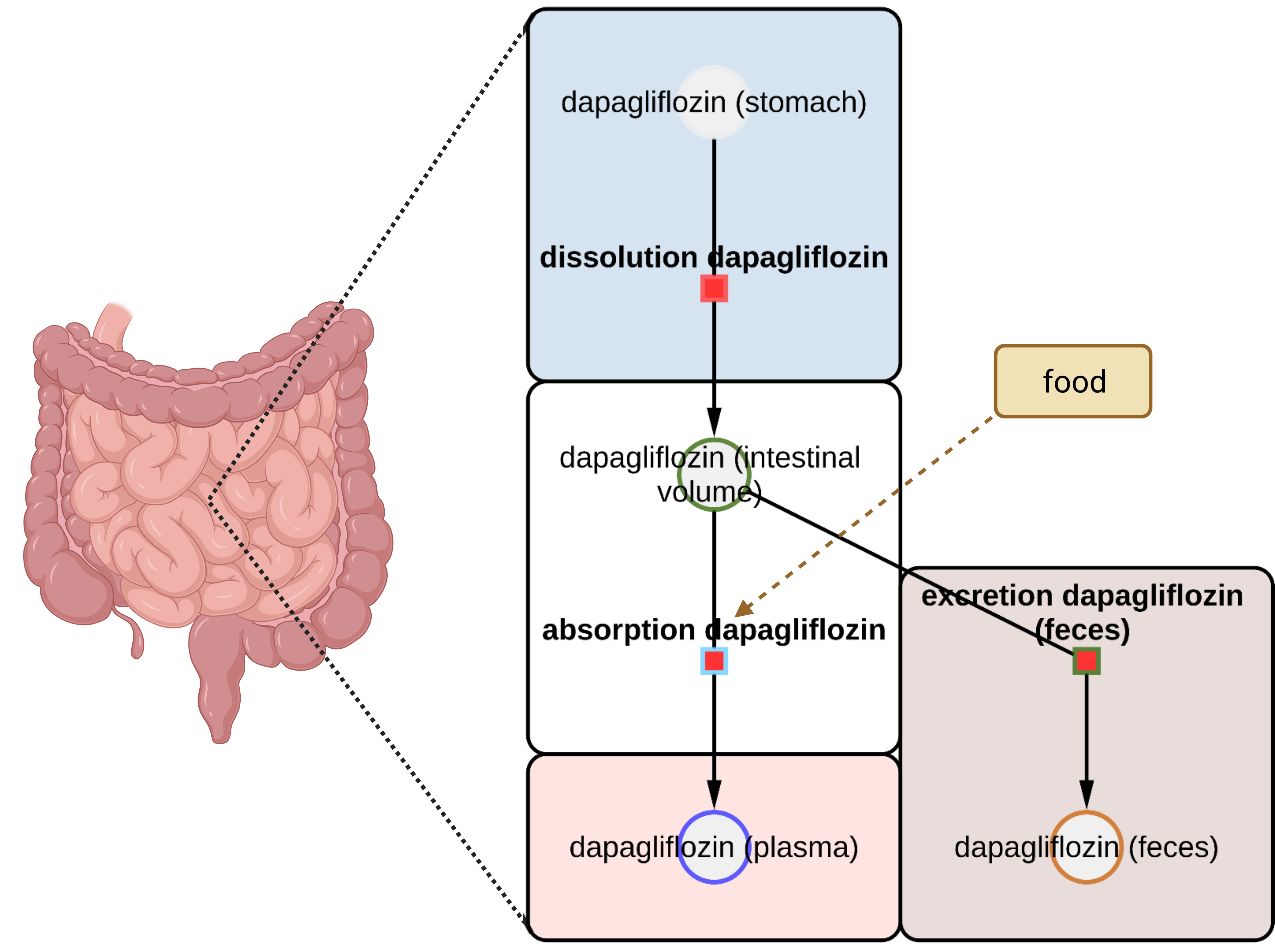

Supplement: Supplementary file 1 [file pharmaceutics-18-00287-s001.zip › Figures/FigS02_intestine_model.png]

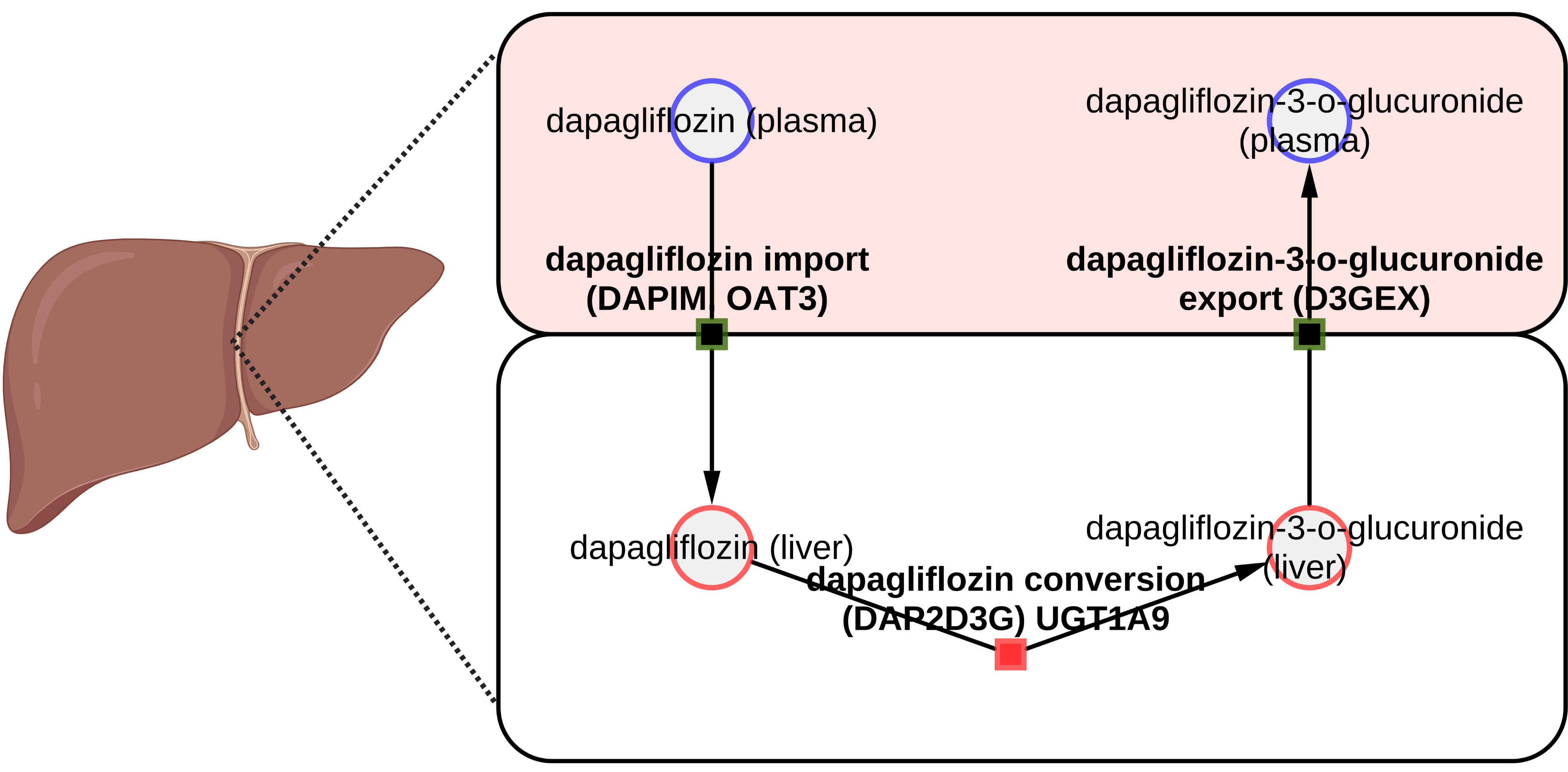

Supplement: Supplementary file 1 [file pharmaceutics-18-00287-s001.zip › Figures/FigS03_liver_model.png]

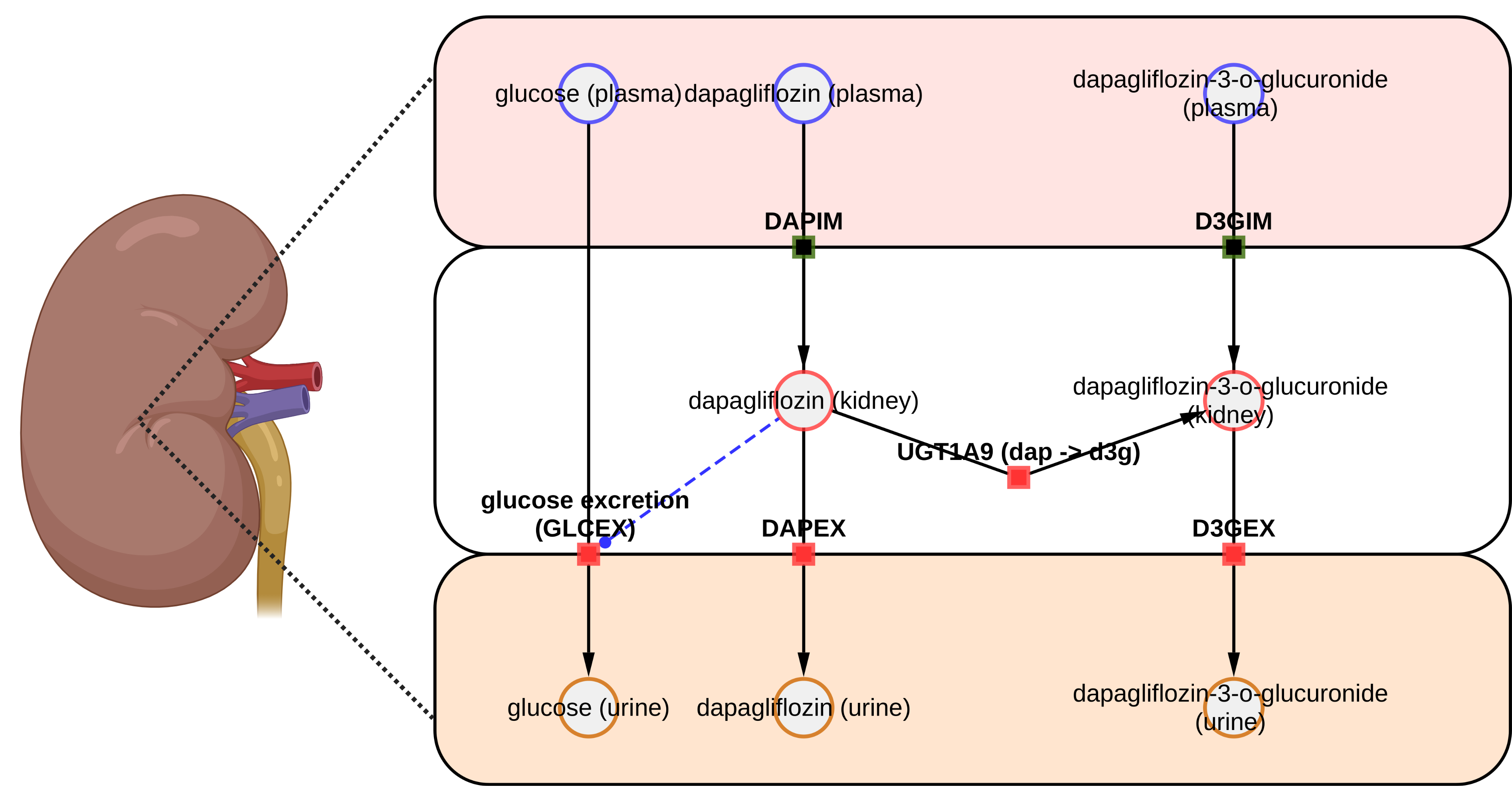

Supplement: Supplementary file 1 [file pharmaceutics-18-00287-s001.zip › Figures/FigS04_kidney_model.png]

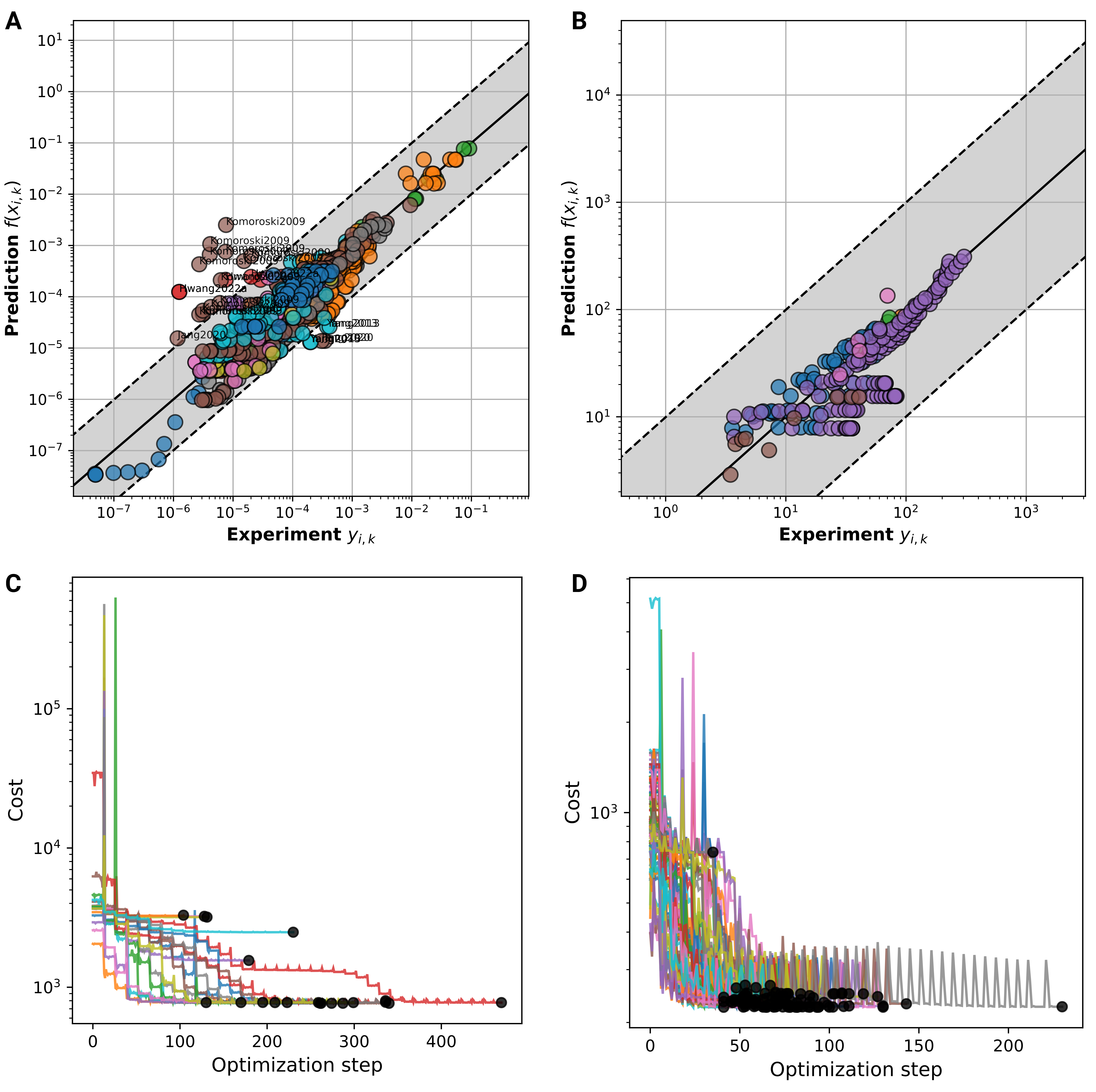

Supplement: Supplementary file 1 [file pharmaceutics-18-00287-s001.zip › Figures/FigS05_fitting.png]

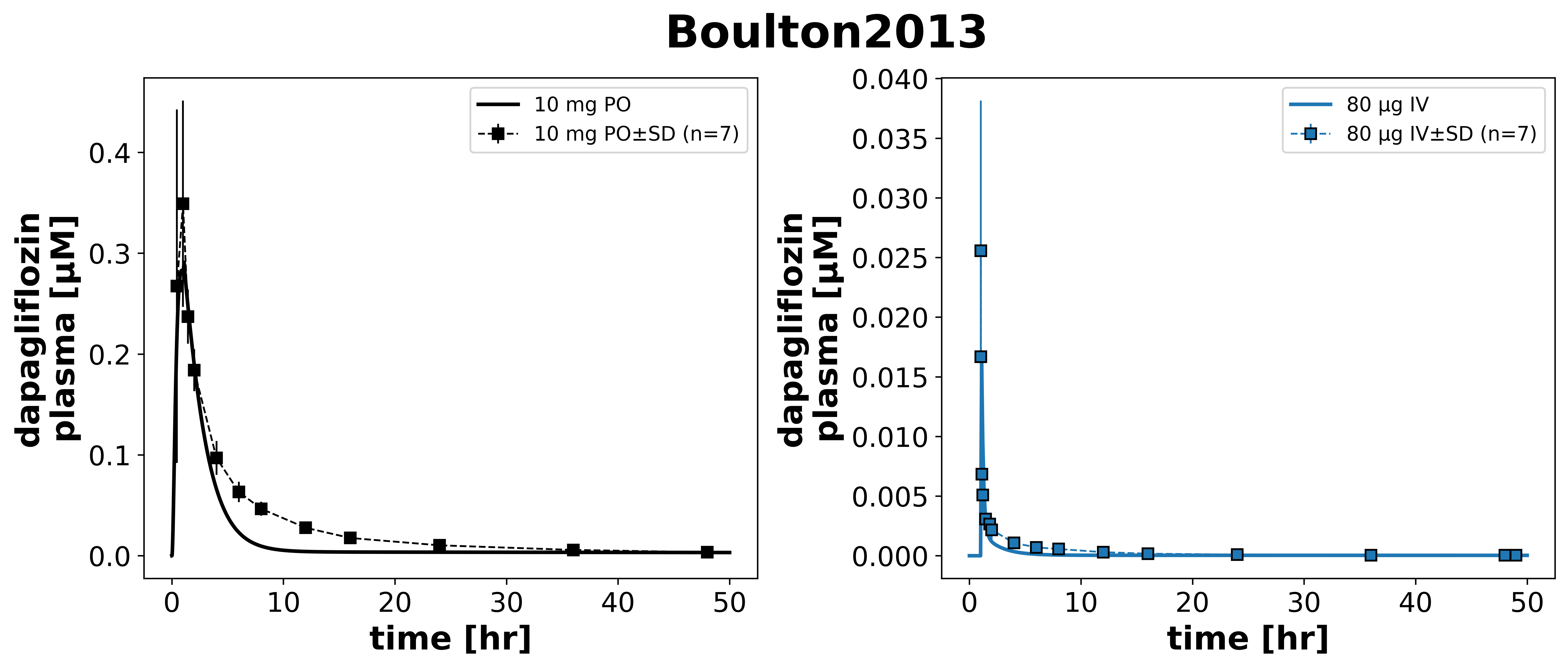

Supplement: Supplementary file 1 [file pharmaceutics-18-00287-s001.zip › Figures/FigS06_Boulton2013_Fig1.png]

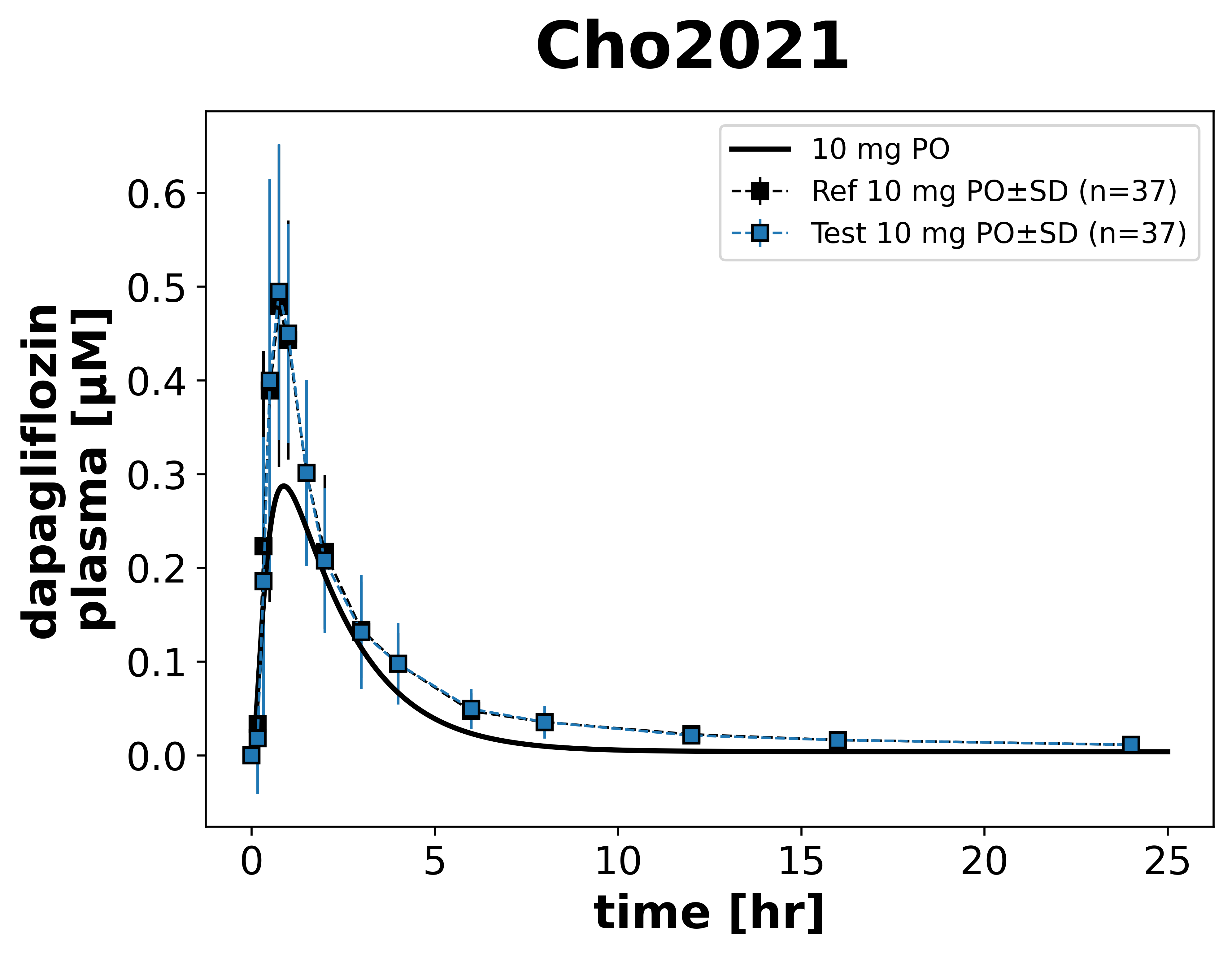

Supplement: Supplementary file 1 [file pharmaceutics-18-00287-s001.zip › Figures/FigS07_Cho2021_Fig6.png]

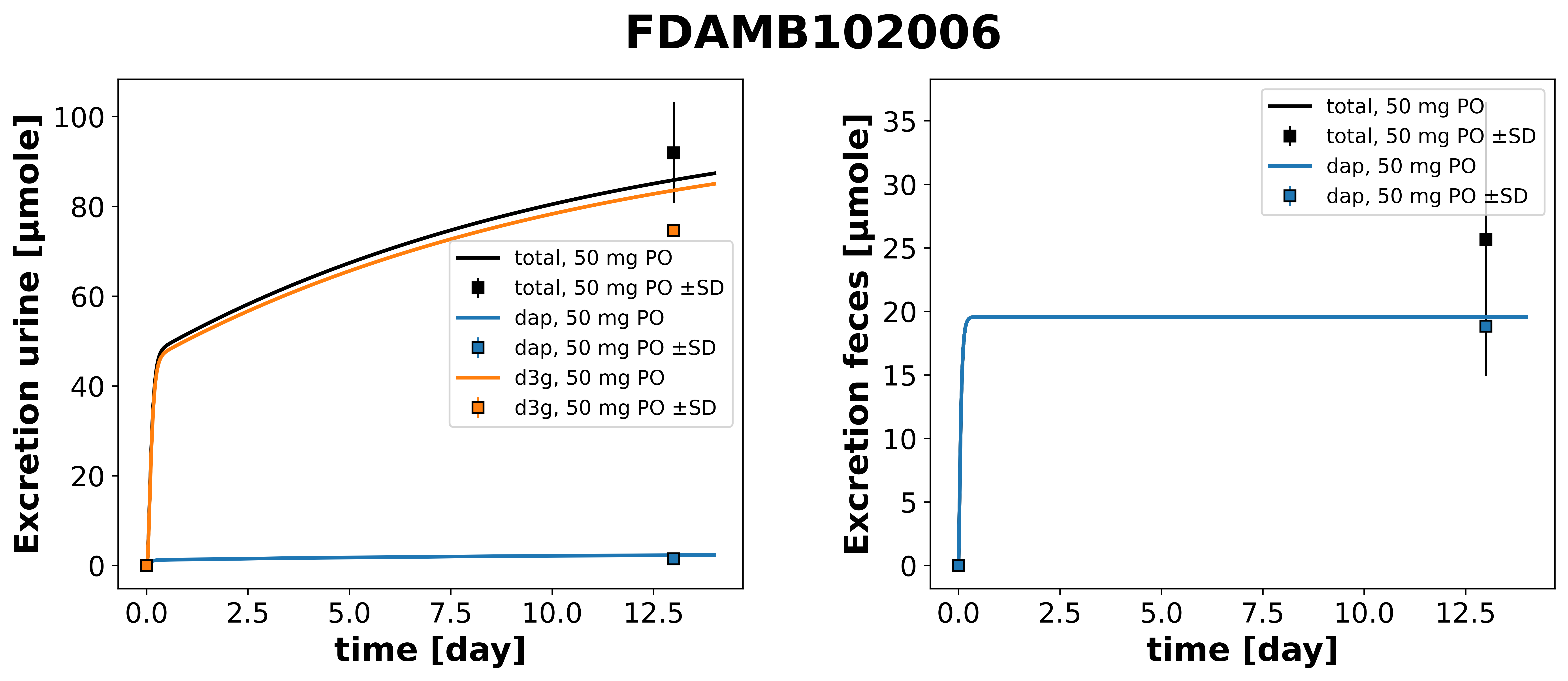

Supplement: Supplementary file 1 [file pharmaceutics-18-00287-s001.zip › Figures/FigS08_FDAMB102006_Tab11.png]

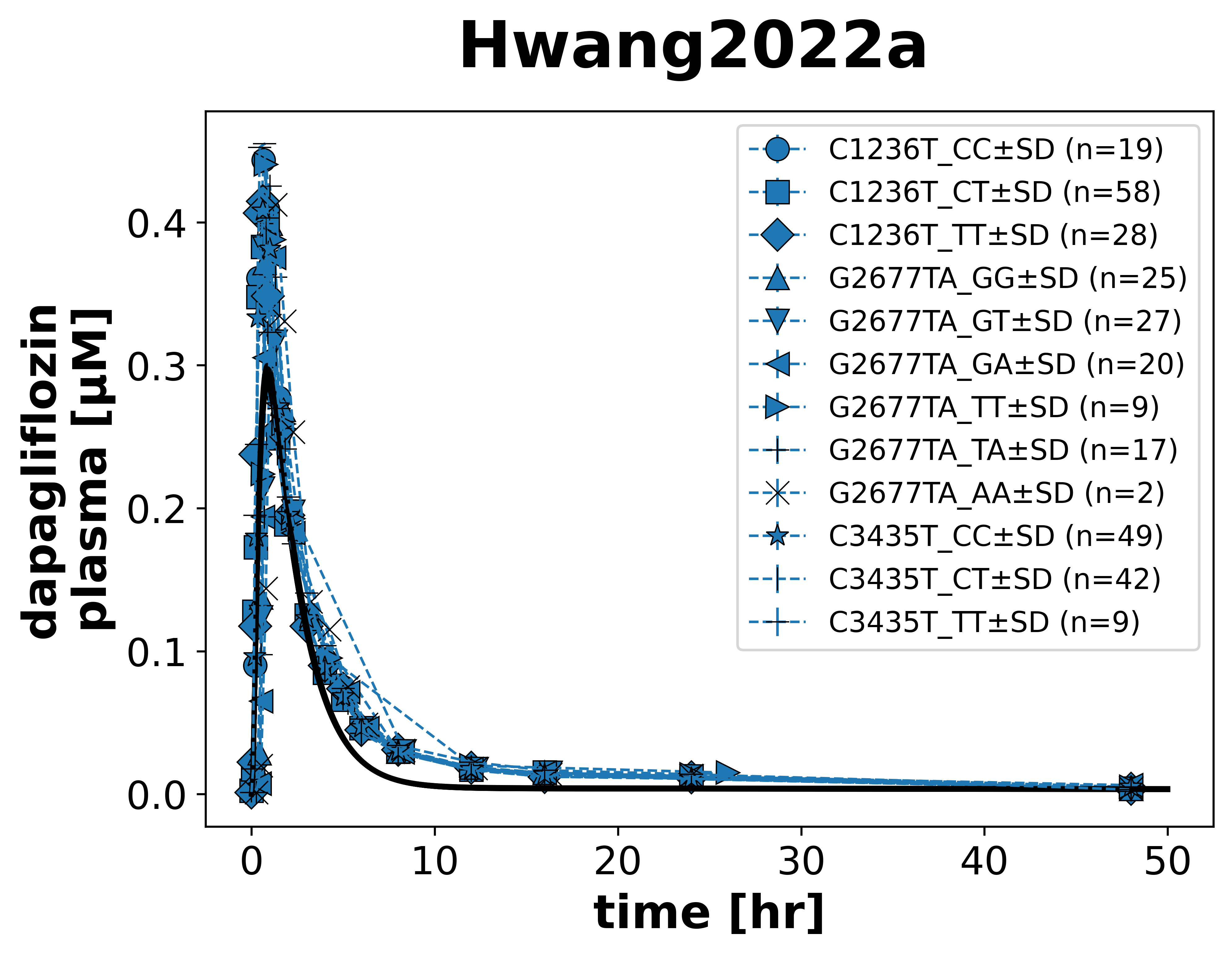

Supplement: Supplementary file 1 [file pharmaceutics-18-00287-s001.zip › Figures/FigS09_Hwang2022a_Fig2.png]

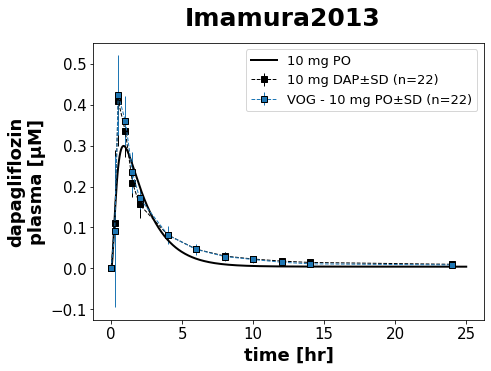

Supplement: Supplementary file 1 [file pharmaceutics-18-00287-s001.zip › Figures/FigS10_Imamura2013_Fig2.png]

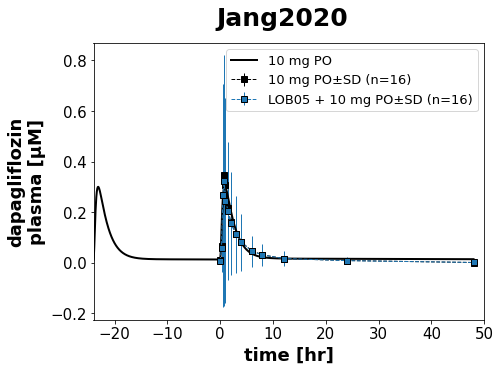

Supplement: Supplementary file 1 [file pharmaceutics-18-00287-s001.zip › Figures/FigS11_Jang2020_Fig2.png]

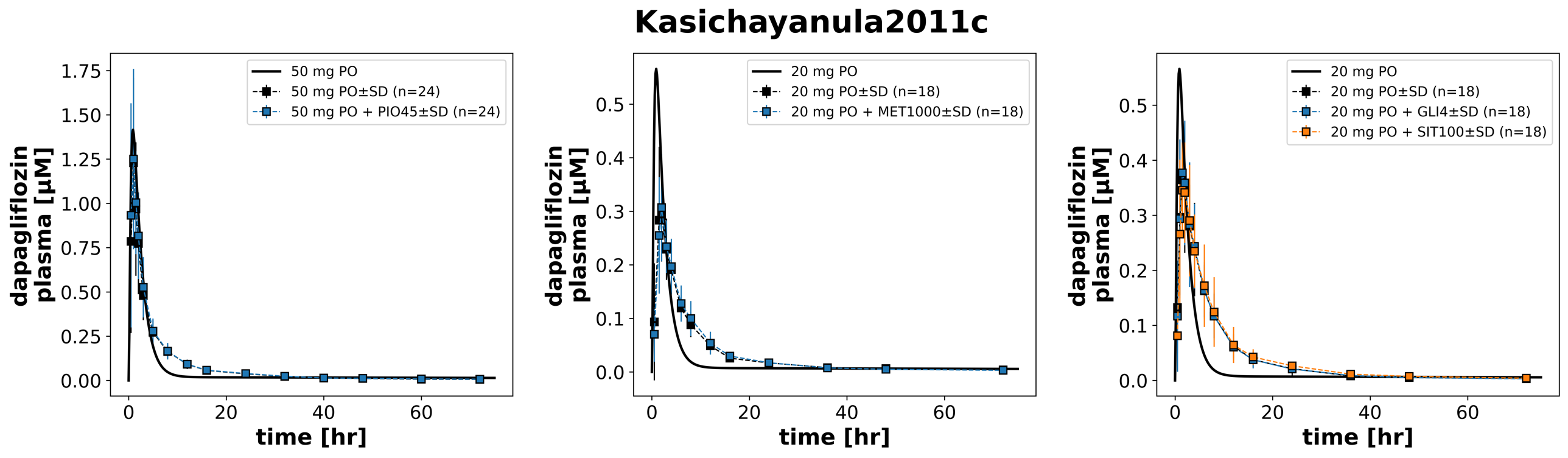

Supplement: Supplementary file 1 [file pharmaceutics-18-00287-s001.zip › Figures/FigS12_Kasichayanula2011c_Fig3.png]

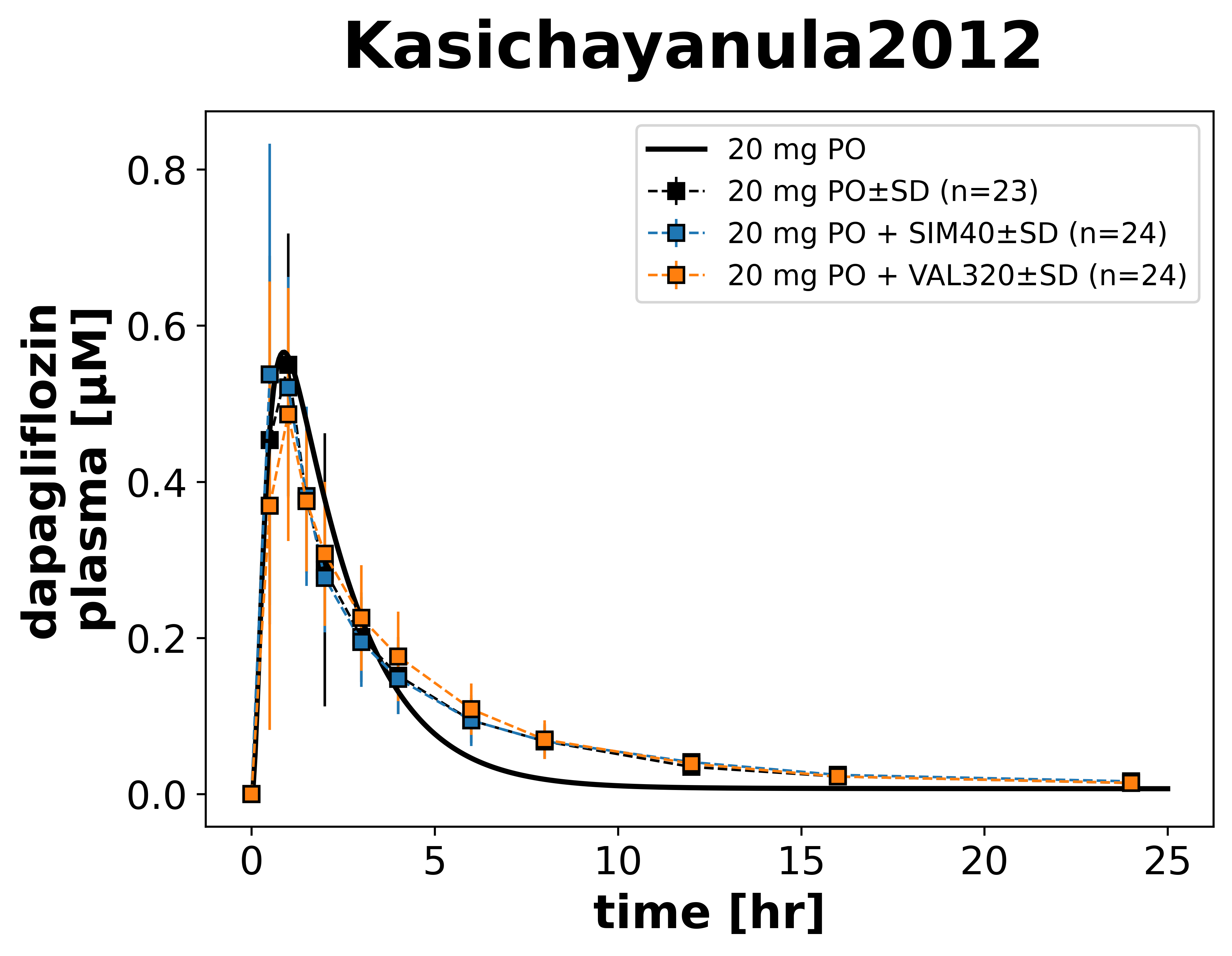

Supplement: Supplementary file 1 [file pharmaceutics-18-00287-s001.zip › Figures/FigS13_Kasichayanula2012_Fig2.png]

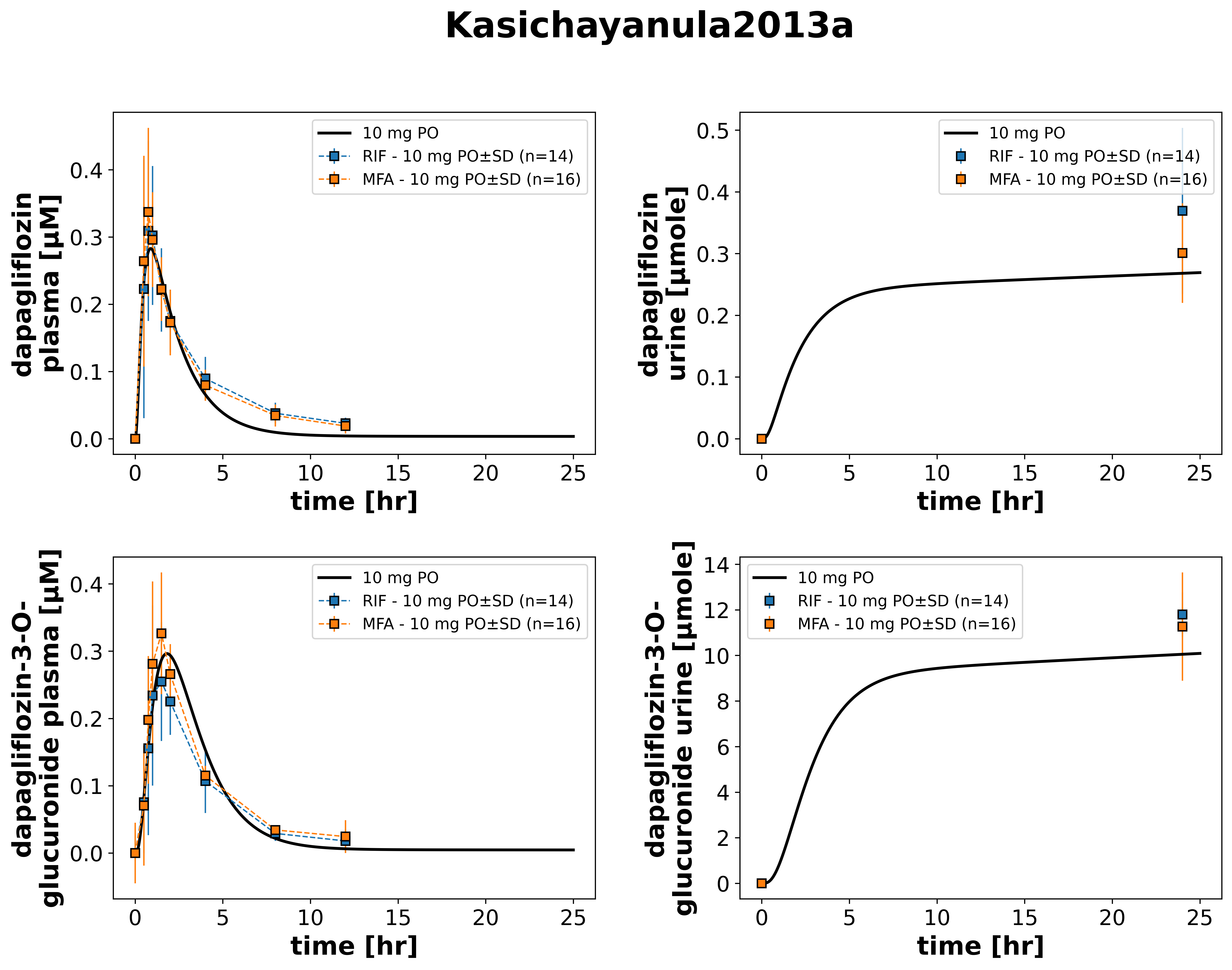

Supplement: Supplementary file 1 [file pharmaceutics-18-00287-s001.zip › Figures/FigS14_Kasichayanula2013a_Fig1.png]

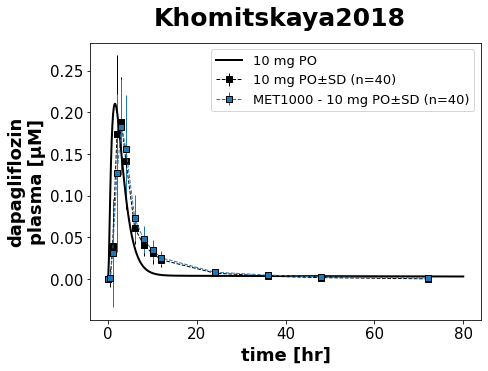

Supplement: Supplementary file 1 [file pharmaceutics-18-00287-s001.zip › Figures/FigS15_Khomitskaya2018_Fig2.png]

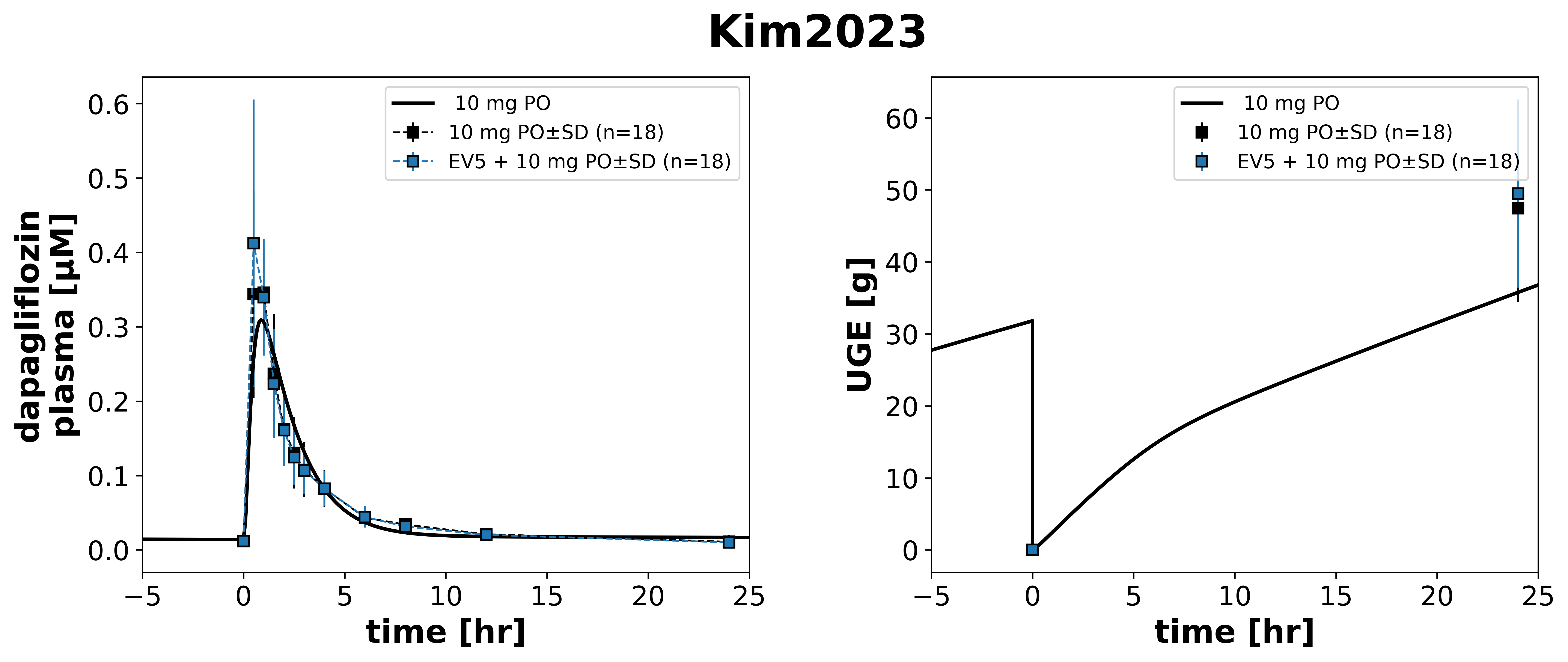

Supplement: Supplementary file 1 [file pharmaceutics-18-00287-s001.zip › Figures/FigS16_Kim2023_Fig2_S2.png]

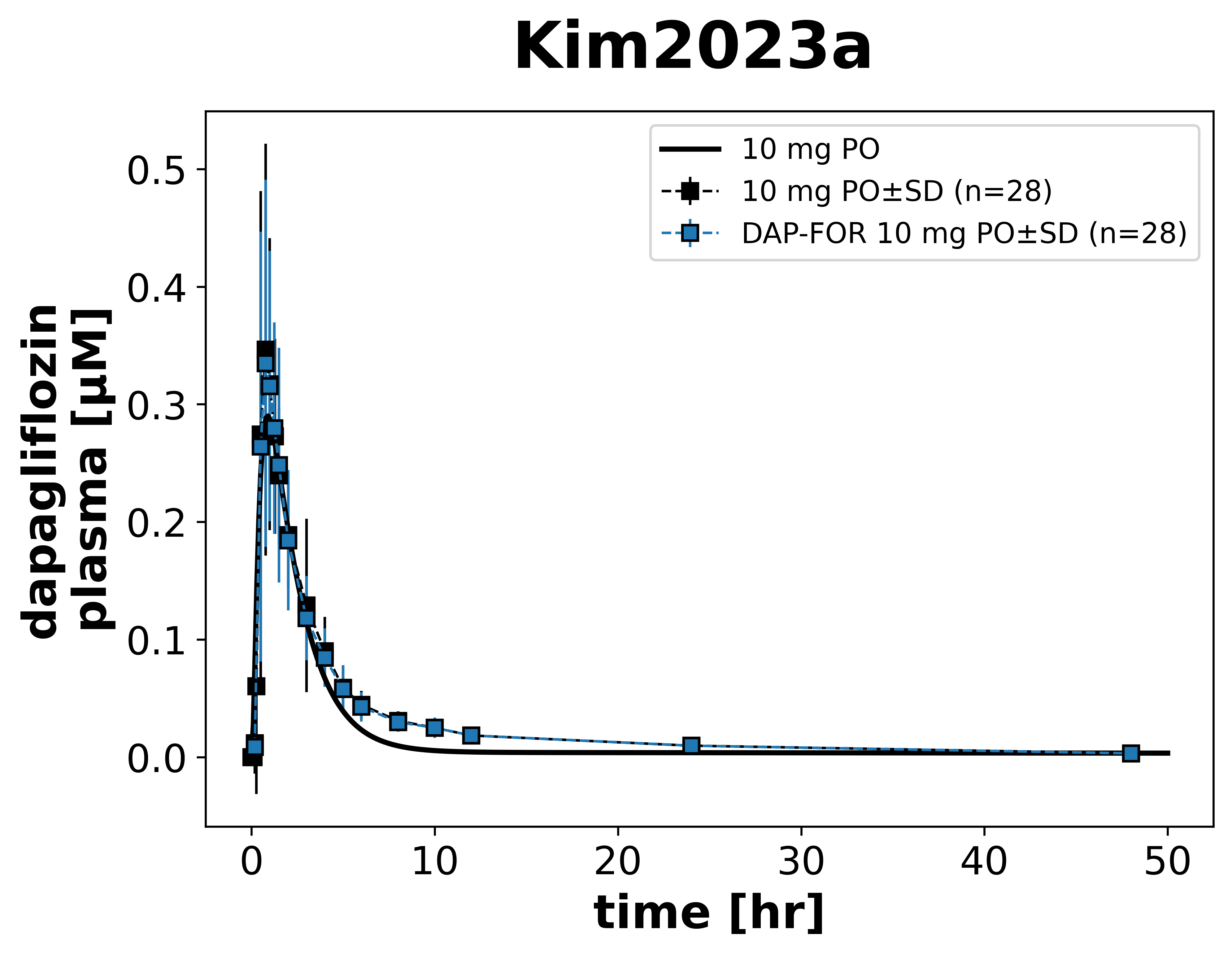

Supplement: Supplementary file 1 [file pharmaceutics-18-00287-s001.zip › Figures/FigS17_Kim2023a_Fig2.png]

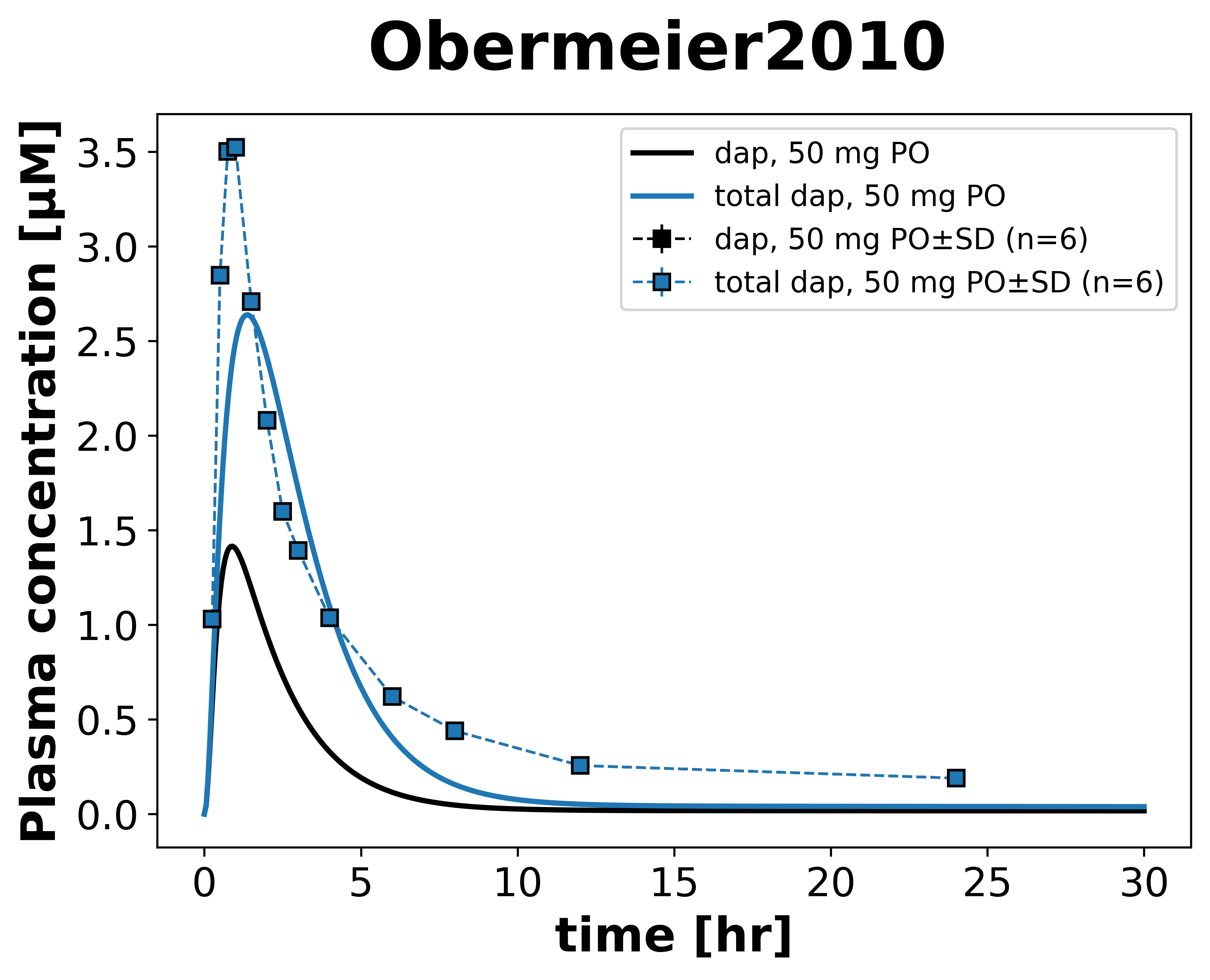

Supplement: Supplementary file 1 [file pharmaceutics-18-00287-s001.zip › Figures/FigS18_Obermeier2010_Fig6.png]

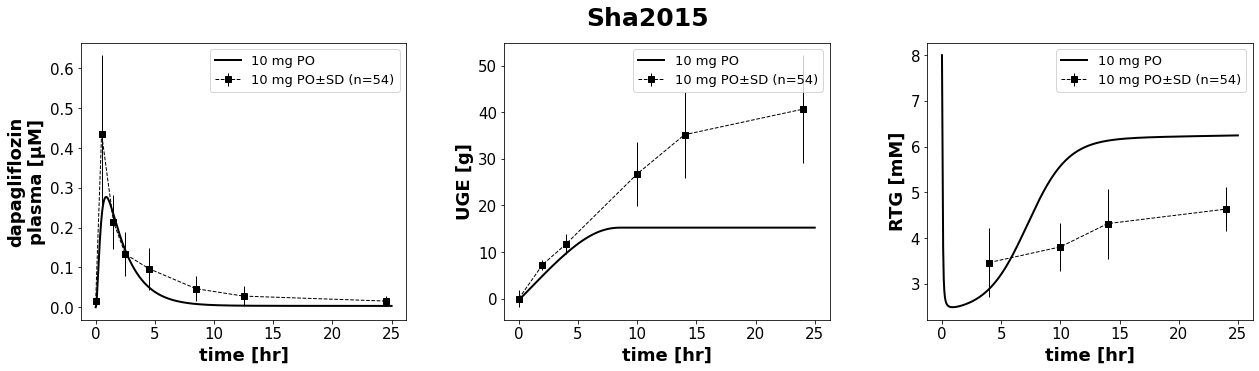

Supplement: Supplementary file 1 [file pharmaceutics-18-00287-s001.zip › Figures/FigS19_Sha2015.png]

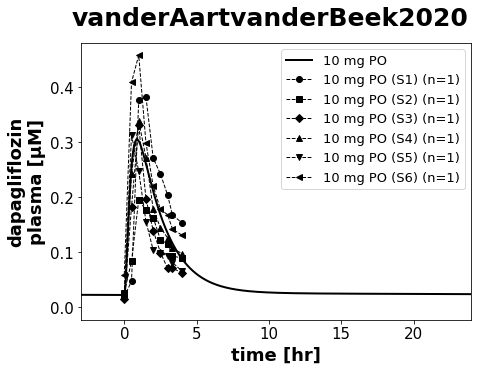

Supplement: Supplementary file 1 [file pharmaceutics-18-00287-s001.zip › Figures/FigS20_vanderAartvanderBeek2020_Fig3.png]

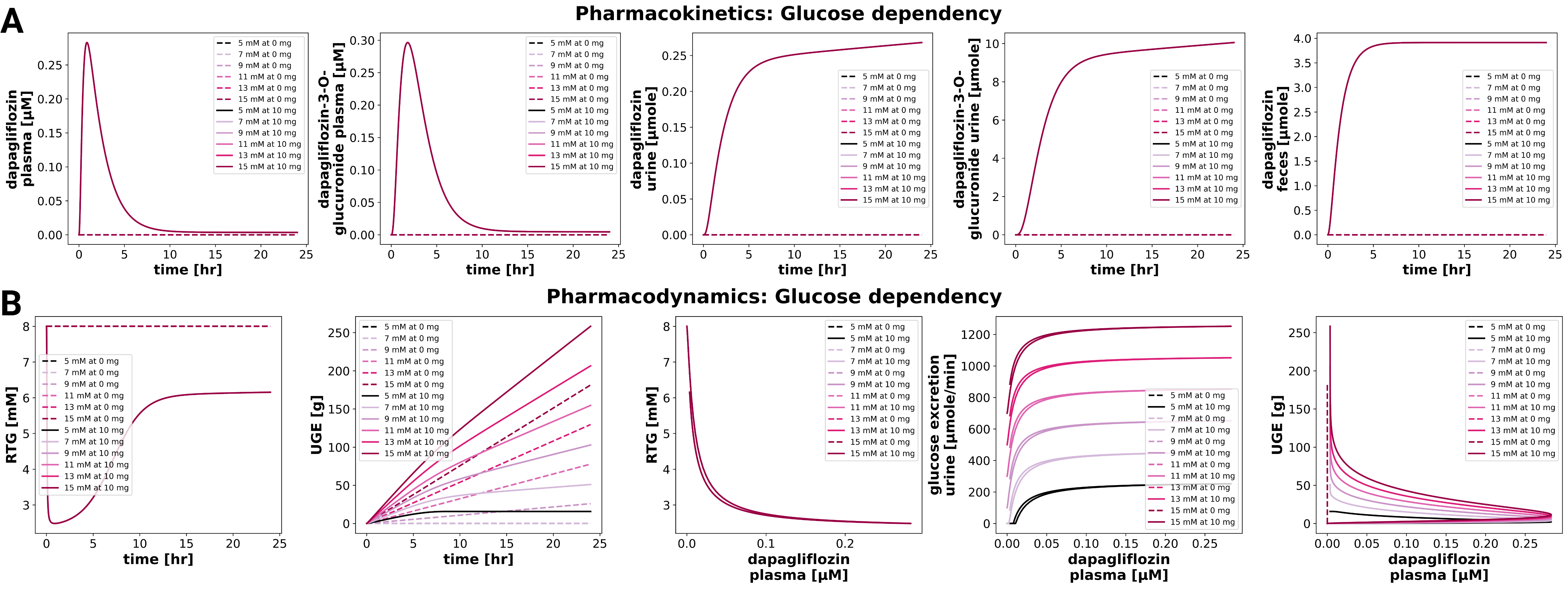

Supplement: Supplementary file 1 [file pharmaceutics-18-00287-s001.zip › Figures/FigS21_glucose_dependency.png]

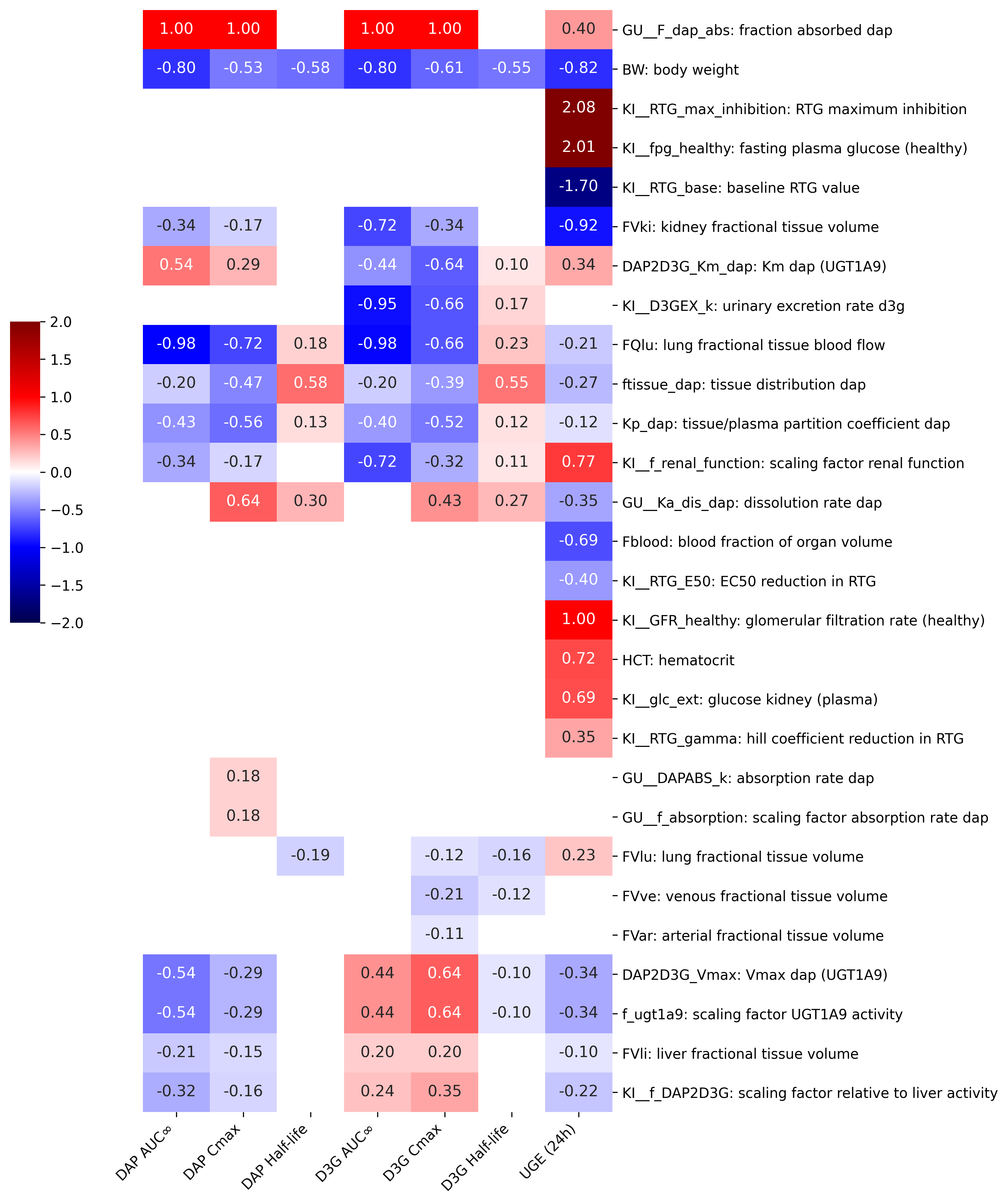

Supplement: Supplementary file 1 [file pharmaceutics-18-00287-s001.zip › Figures/FigS22_local_sensitivity.png]
